# Methanol fixed feeder layers altered the pluripotency and metabolism of bovine pluripotent stem cells

**DOI:** 10.1038/s41598-022-13249-3

**Published:** 2022-06-02

**Authors:** Wenqiang Xu, Ruifeng Hao, Jing Wang, Lingna Gao, Xuejie Han, Chen Li, Shu Fang, Hui Zhang, Xueling Li

**Affiliations:** 1 State Key Laboratory of Reproductive Regulation and Breeding of Grassland Livestock, School of Life Sciences, Inner Mongolia University, Hohhot, People’s Republic of China; 2 Inner Mongolia Key Laboratory of Hypoxic Translational Medicine, Baotou Medical College, Baotou, Inner Mongolia People’s Republic of China; 3 School of Biomedical and Pharmaceutical Sciences, Guangdong University of Technology, Guangzhou, People’s Republic of China

**Keywords:** Biological techniques, Stem cells

## Abstract

The pluripotency maintenance of pluripotent stem cells (PSCs) requires the suitable microenvironment, which commonly provided by feeder layers. However, the preparation of feeder layers is time consuming and labor exhaustive, and the feeder cells treated with mitomycin C or γ-ray irradiation bring heterologous contamination. In this study, mouse embryonic fibroblasts (MEFs) were treated by methanol to generate chemical fixed feeder cells, and bovine embryonic stem cells F7 (bESC-F7) cultured on this feeder layer. Then the pluripotency and metabolism of bESC-F7 cultured on methanol-fixed MEFs (MT-MEFs) named MT-F7 was compared with mitomycin C treated MEFs (MC-MEFs). The results showed that bESC-F7 formed alkaline phosphatase positive colonies on MT-MEFs, the relative expression of pluripotent markers of these cells was different from the bESCs cultured on the MC-MEFs (MC-F7). The long-term cultured MT-F7 formed embryoid bodies, showed the ability to differentiate into three germ layers similar to MC-F7. The analyses of RNA-seq data showed that MT-MEFs lead bESCs to novel steady expression patterns of genes regulating pluripotency and metabolism. Furthermore, the bovine expanded pluripotent stem cells (bEPSCs) cultured on MT-MEFs formed classical colonies, maintained pluripotency, and elevated metabolism. In conclusion, MT-MEFs were efficient feeder layer that maintain the distinctive pluripotency and metabolism of PSCs.

## Introduction

Pluripotent stem cells (PSCs) derived from the inner cell mass of preimplantation blastocysts or induced by pluripotent factors possess the unlimited potential for self-renewal and the ability to differentiate into multiple somatic cell types^1,2^. Recently, the establishment of naïve pluripotent stem cells and expanded pluripotent stem cells (EPSCs) contributing to both the embryonic and extraembryonic cell lineages in humans, mice, pigs, and cattle has been reported^3–7^. Stable bovine PSC lines are beneficial for studying the mechanisms underlying the pluripotency of domestic animal and very useful in the animal husbandry for genomic selection, gene editing, and production of cattle with high genetic values. Although the feeder-free culture systems established, the feeder layer still has unique advantages in the long-term stable culture of PSCs. Studies has shown that the feeder layers secrete essential nutrients, growth factors, and cytokines that are essential for the self-renewal and pluripotency maintenance of PSCs^8–12^. However, it has also been reported that some of these soluble factors secreted by MEFs are not necessary for maintaining PSCs^13^. The feeder cells also generate extracellular matrix (ECM) proteins that interact with the integrins on the plasma membrane of PSCs, which promote cell adhesion and activate intracellular signaling pathways that are essential for self-renewal and maintenance of PSCs^14–16^. The chemically fixed ECM treated with collagenase not only lost the original surface structure, but also lost the ability to promote the adhesion and proliferation of PSCs^13^.

MEFs treatment with mitomycin C or γ-ray irradiation to arrest their growth and mitosis commonly used as feeder cells for PSCs. However, these MEFs consume nutrients and release metabolic waste into the PSC medium, which may affects the growth and maintenance of PSCs. Moreover, these MEFs are sensitive to drugs for selecting transformed PSCs, unless MEFs were previously transfected by drug-resistant genes^13^. In addition, although feeder-free culture provides a promising alternative, it requires a longer time for colony formation^17,18^ and may induce karyotype instability in the PSCs^19^.

The feeder layers prepared by chemical fixation maintain long-term self-renewal and differentiation potential of mouse and human PSCs^13,18,20^. The advantages to use chemical-fixed feeder cells consist of: (1) the preparation is easy and cost and labor effective; (2) the termination of metabolism of feeder cells leads to no competition for nutrients and oxygen with PSCs; (3) supplying the ready-to-use feeder cells; (4) fixed feeder cells are resistant to drugs and allow to rapidly screening of stably transfected PSCs. On top of the traditional PSC culture based on mitotically inactivated feeders^21,22^ and stem cell culture without feeder layer^23–27^, the chemically fixed feeder layer provides an alternative source for culturing or maintaining of PSCs^28–30^.

PSCs have the unique gene expression patterns and metabolic characteristics, and the maintenance of pluripotent state is related to the activities of important metabolic pathways such as glycolysis and fatty acid de novo biosynthesis^31–33^. The changes in the microenvironment induce differences in the gene expression patterns of PSCs that may affect their pluripotency and differentiation potentials. Previous studies focused on the maintenance of pluripotency and differentiation potential of the PSCs grown on the feeder layers treated with chemical reagents^13,18,20^. In this study, we analyzed the pluripotency and metabolism of PSCs on chemical-fixed feeder cells from the perspective of global gene expression profile and metabolic characteristics, which will provide a reference for the optimization of the culture system for PSCs.

## Materials and methods

### Animals

Using C57 mice to make mouse embryonic fibroblasts (MEFs) was approved by the Animal Care and Use Committee of Inner Mongolia University. The experimental procedures that are involved in animal samples based on the guidelines and regulations approved by the Animal Research Committee of Inner Mongolia University. All methods are reported in accordance with ARRIVE guidelines (https://arriveguidelines.org) for the reporting of animal experiments.

### Feeder layer preparation

The mitomycin C treated MEFs (MC-MEFs) were prepared as traditional procedures: Primary MEFs from C57 mice were thawed and cultured at 37 °C and 5% CO_2_ in DMEM medium supplemented with 10% fetal bovine serum and 1% penicillin/streptomycin. The 3rd passage primary MEFs at 90% confluence were treated with 12 μg/mL mitomycin C for 3 h, and then preserved in liquid nitrogen. The feeder MEFs were thawed quickly, seeded in petri dishes coated with gelatin and cultured in DMEM medium at 37 °C and 5% CO_2_ for 12–24 h. Then, MEFs were washed with Dulbecco’s Phosphate-Buffered Saline (DPBS) and replaced with bESC culture medium before plate bESCs.

The methanol fixed MEFs (MT-MEFs) were prepared as follows: MEFs grown in DMEM medium until they reached 90% confluence in 35-mm petri dishes (Corning, USA). Then, the MEFs were washed with DPBS, and submerged in pure methanol (prechilled at 4 °C) at room temperature for 10 min. Then, after removing the methanol, the dried MT-MEFs were stored at room temperature, rinsed with DPBS, and replaced with bESC medium before bESCs culture.

The petri dishes with MT-MEFs could reused up to 3 times. After the stem cells were dissociated, the dishes were washed twice with DPBS to remove the floating residues, and dried in a ventilated place with the lids opening, then cover the lids and sealed with a sealing film to prevent moisture absorption before storage (at 4 °C).

### Culturing of bovine ESCs on MC-MEFs and MT-MEFs

The bESC-F7 cells (abbreviated as F7) was established from in vitro fertilized (IVF) bovine embryos as previously described^34^. The culture medium comprised of the Customized mTeSR1 basal medium (free of growth factors, Stem cell), recombinant human fibroblast growth factor (rhFGF, 20 ng/mL, Peprotech, USA) and classical WNT signaling pathway inhibitor IWR1 (2.5 μM, Sigma Aldrich, USA), as previously described (CTFR)^35^.

The F7 cells grown on MC-MEFs at 37 °C and 5% CO_2_, were named as MC-F7. The culture medium was refreshed every day. The cells were dissociated with TrypLE (Gibco, USA) when they reached about 70% confluence (at 5th days). The fresh medium supplemented with 10 μM ROCK inhibitor Y-27632 to improve cell viability. The dissociated cells were placed in a 100-mm gelatin-coated petri dish for 25 min. The floating cells were collected for real-time quantitative polymerase chain reaction (RT-PCR), western blotting, and RNA-seq experiments.

The F7 cells cultured with MT-MEFs at 37 °C and 5% CO_2_, were named as MT-F7. The culture medium was refreshed every other day. The culture conditions and other procedures were same as those used for the MC-F7 cells.

### Alkaline phosphatase staining

Alkaline phosphatase (AP) staining was performed using the BCIP/NBT AP chromogenic kit (Beyotime, China). Briefly, the stem cells were washed twice with prechilled DPBS at 4 °C, and then fixed with 4% paraformaldehyde (Solarbio, China) at 21–26 °C for 15 min. The cells were then incubated with the staining solution for 30 min in the dark at room temperature. After rinsing twice with DPBS, the cells were photographed using an inverted light microscope (Nikon, Japan).

### Growth curve of bESCs

The CCK-8 cell count kit (Solarbio, China) was used to evaluate the growth rate of bESCs. In brief, 3 × 10^3^ cells were passaged onto MC-MEFs or MT-MEFs in a 48-well plate. The MC-MEFs or MT-MEFs in CTFR medium were used as parallel blank controls. Then, 20 μl of CCK-8 reagent was added into each well and incubated at 37 °C and 5% CO_2_ for 3 h. The absorbance was measured at 450 nm every 24 h for 3 days. The growth rate was calculated using the ratio of OD value at D (n)/D (n − 1).

### RT-PCR analysis

Total RNA was extracted from the cultured PSCs using the Arcturus PicoPure RNA Isolation Kit (Thermo Fisher Scientific, USA) and reverse transcribed with the PrimeScript RT Reagent Kit with gDNA Eraser (Takara, Japan) according to the manufacturers protocols. Then, quantitative real-time polymerase chain reaction (RT-PCR) was performed using the KAPA SYBR FAST Universal RT-PCR master mix (Kapa Biosystems, South Africa) according to the manufacturers protocol. The RT-PCR primers were designed using the Primer Premier 5 software (Premier, Canada) and their specificity was tested using Primer-BLAST (https://www.ncbi.nlm.nih.gov/tools/primer-blast/). Then, the primers were synthesized by BGI Co., Ltd (China). The primer sequences are shown in Supplementary Table S1. The gene expression levels were determined using the 2^−△△CT^ method relative to the house keeping genes such as HMBS^36^, or ACTB.

### Western blotting

Total protein lysates were prepared from cells by incubating for30min with the Mammalian Protein Extraction Reagent (Beyotime, China) and PMSF (Beyotime, China) protease inhibitor at 4 °C. The protein extracts were centrifuged at 14,600×*g* for 5 min. The supernatants were transferred into a clean tube and centrifuged at 14,600×*g* for 5 min. Then, protein lysates were mixed with 5× protein loading buffer, boiled at 100 °C for 5 min, and incubated on ice for 5 min. The SDS–polyacrylamide gel electrophoresis of protein ladder (Thermo Fisher, USA), internal reference proteins and target proteins was performed at the same time, and the complete gels including visible protein ladder with colored bands and invisible undeveloped blots. Before hybridizing with antibodies, the full-length gel according to the molecular weight of the target proteins was cut and ensured including the target proteins. The separated proteins were transferred onto polyvinylidene fluoride (PVDF) membrane (Millipore, USA) which then blocked with 5% skimmed milk in 1× TBST. Then, the blots were incubated with primary antibodies (anti-SOX2 (1:100, Cell Signaling Technology, USA), anti-OCT4 (1:100, Santa Cruz Biotechnology, USA), anti-CDX2 (1:100, Cell Signaling Technology, USA), anti-β-Actin (1:100, Boster, USA), anti-GAPDH (1:100, Cell Signaling Technology, USA)) overnight at 4 °C. The blots were then incubated with secondary antibodies, goat anti-rabbit (1:500, Abcam, UK), or goat anti-mouse (1:500, Millipore, USA) for 1 h at 37 °C. The blots were developed with ECL chemiluminescence reagent (CWBio, China) and photographed using the Chemiluminescence imager (Tanon-5200, China).

### Immunofluorescence staining

The cells were fixed with 4% paraformaldehyde (PFA, Solarbio, China) for 10 min at room temperature, permeabilized with DPBS containing 1% (v/w) Triton X 100 (Solarbio, China) for 30 min, and blocked with 10% (V/V) goat serum in DPBS for 1 h. After washing, the cells were incubated overnight at 4 °C with following antibodies in blocking buffer: anti-SOX2 (1:100, Cell Signaling Technology, USA), anti-OCT4 (1:100, Santa Cruz Biotechnology, USA), anti-NANOG (1:100, Peprotech, USA), anti-CDX2 (1:100, Cell Signaling Technology, USA), anti-SMA (1:100, Abcam, UK), anti-AFP (1:100, Abcam, UK), and anti-GFAP (1:100, Dako, Denmark) antibodies. Then, after washing thrice with DPBS, the cells were incubated for 1.5 h at 21–26 °C with the following secondary antibodies (diluted 1:500 in the blocking buffer), goat anti-rabbit (Abcam, UK), goat anti-mouse (Millipore, USA), and goat anti-rabbit IgG (Life Technologies, USA). The negative control cells were directly incubated with the secondary antibodies to eliminate any false positives. Then the samples were stained with DAPI (0.5 mg/mL, Beyotime, China) in DPBS for 5 min. Then, the cells were washed thrice with DPBS and mounted on glass slides with the anti-fading solution (Solarbio, China). The fluorescent signals of the samples were visualized using the Nikon A1 confocal laser-scanning microscope (Nikon, Japan).

### In vitro differentiation of bovine ESCs on MC-MEFs and MT-MEFs

The bESCs were cultured with the first-stage differentiation medium (90% IMDM (Thermo Fisher Scientific, USA) plus 10% FBS) for 6–7 days. Fresh medium was added every 2 days. Then, the embryoid bodies were suspended in the second-stage differentiation medium (90% DMEM plus 10% FBS) and transferred to glass slides that incubated with gelatin for adherent growth. The differentiation medium was changed every day. After 3 weeks, the differentiated cells were subjected to immunofluorescence staining as described in the previous section with antibodies against SMA, AFP, and GFAP to determine the differentiation status.

### RNA sequencing analysis

Total RNA was extracted by the Arcturus™ PicoPure™ RNA Isolation Kit (Thermo Fisher Scientific, USA) according to the provided procedure. NanoDrop 2000c Spectrophotometer (Thermo Fisher Scientific, USA) was used to determine the quality and quantity of RNA. 1 mg of total RNA and the PrimeScript™ RT Reagent Kit with gDNA Eraser (Takara, Japan) were applied to reverse transcription and cDNA synthesis. The RNA sequencing library was prepared using Illumina NEBNext Ultra RNA Library Prep Kit (NEB, USA) according to the manufacturers protocols. Then, the library quality was assessed and 150 bp paired-end reads were generated by Illumina sequencing. The image data from the sequenced fragments was processed through a high-throughput sequencer and converted into sequence data in the fastq format (reads). High quality data was obtained after filtering. The reference genome and gene model annotation files were downloaded from the open genome website (Ensembl). Hisat2 v2.0.5. Software (http://daehwankimlab.github.io/hisat2/) was used to build the index of the reference genome. The clean paired end reads were aligned to the reference genome. The read numbers mapping to each gene were calculated using the Feature Counts v1.5.0-p3 software. TMM (Trimmed Mean of M-values) was employed to standardize the counting data of the reads. EdgeR was used to analyze the differentially expressed genes (DEGs). P-values were adjusted for the false discovery rate (FDR). The genes with fold changes greater than 1 and P-value < 0.05 were marked as differentially expressed. Cluster Profiler was used for Gene Ontology (GO) and KEGG enrichment analysis of differentially expressed genes. The RNA-seq data was converted into TPM and used for analysis of relative gene expression.

### Statistical analysis

The data was represented as the average ± SEM values of at least three technical replicates. The statistical analysis was performed using the GRAPHPAD PRISM 5 software (GraphPad Software Inc., USA). Student’s double-tailed t-test was performed to determine the statistical significance between the groups. P < 0.05 was considered as statistically significant. *denotes P = 0.01–0.05, **denotes P = 0.001–0.01, and ***denotes P < 0.001.

## Results

### The methanol fixed feeder layers maintain the pluripotency and differentiation potential of the bESCs

**Figure 1:**
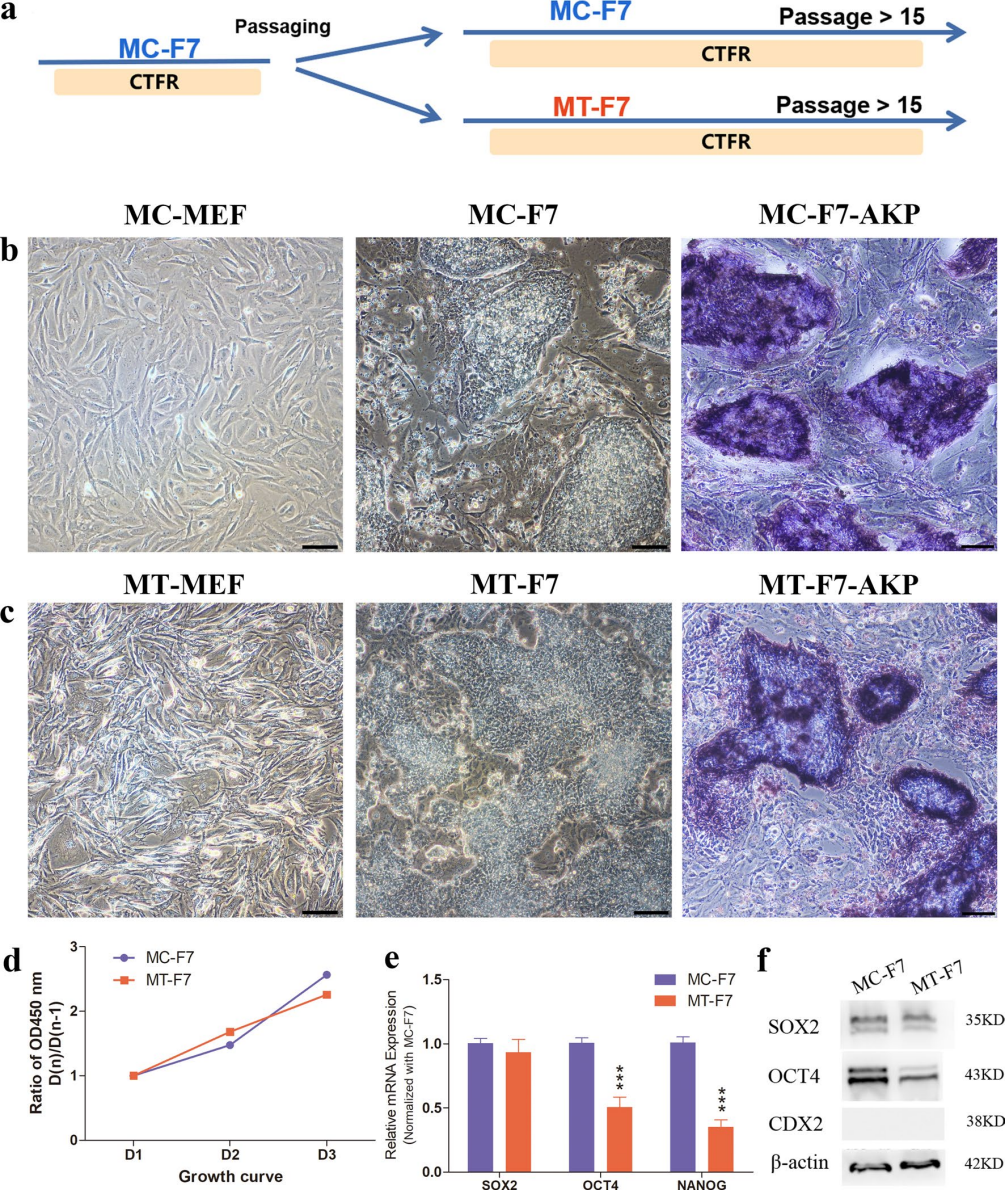
Characterization of bovine ESCs grown on mitomycin C treated and methanol fixed feeder cells. (**a**) Schematic diagram shows the experimental strategy for analyzing bESCs grown on mitomycin C-treated mouse embryonic fibroblasts (MC-MEFs) and methanol-treated MEFs (MT-MEFs). (**b**) The morphology and AKP staining of MC-MEFs and the F7 grown on MC-MEFs (MC-F7). Bar = 100 μm. (**c**) The morphology and AKP staining of MT-MEFs and F7 grown on MT-MEFs (MT-F7). Bar = 100 μm. (**d**) Growth curves of MC-F7 and MT-F7 cells. (**e**) Real-time quantitative polymerase chain reaction (RT-PCR) analysis shows the expression of the key pluripotency regulatory factors OCT4, SOX2, and NANOG in MC-F7 and MT-F7 cells. (**f**) Western blotting analysis shows the protein expression of OCT4, SOX2 and CDX2 in MC-F7 and MT-F7 cells.

To detect the effects of feeder cells on bESCs, we first remove feeder cells from the culture and plate the cells on the gelatin treated plates (feeder-free). When bESCs were transferred from MC-MEFs to feeder-free plate, the attachment of F7in the CTFR system was decreased significantly, and the cells did not survive after passaging. When bESCs were transferred from MC-MEFs to MT-MEFs, the cells easily adapted to the new culture condition. The MT-F7 cells were stable and could be cryopreserved, thawed and resuscitated normally up to 23 passages. The characteristic analyses were carried out after passaged 10 and 15 generations (Fig. 1a). MT-F7 cells were slightly flatter than the MC-F7 cells, but the growth rate and alkaline phosphatase (AKP) staining were comparable (Fig. 1b–d). The morphology of MEFs treated with pure methanol appeared to be wrinkled and dehydrated (Fig. 1c), and can be reused up to three times.

**Figure 2:**
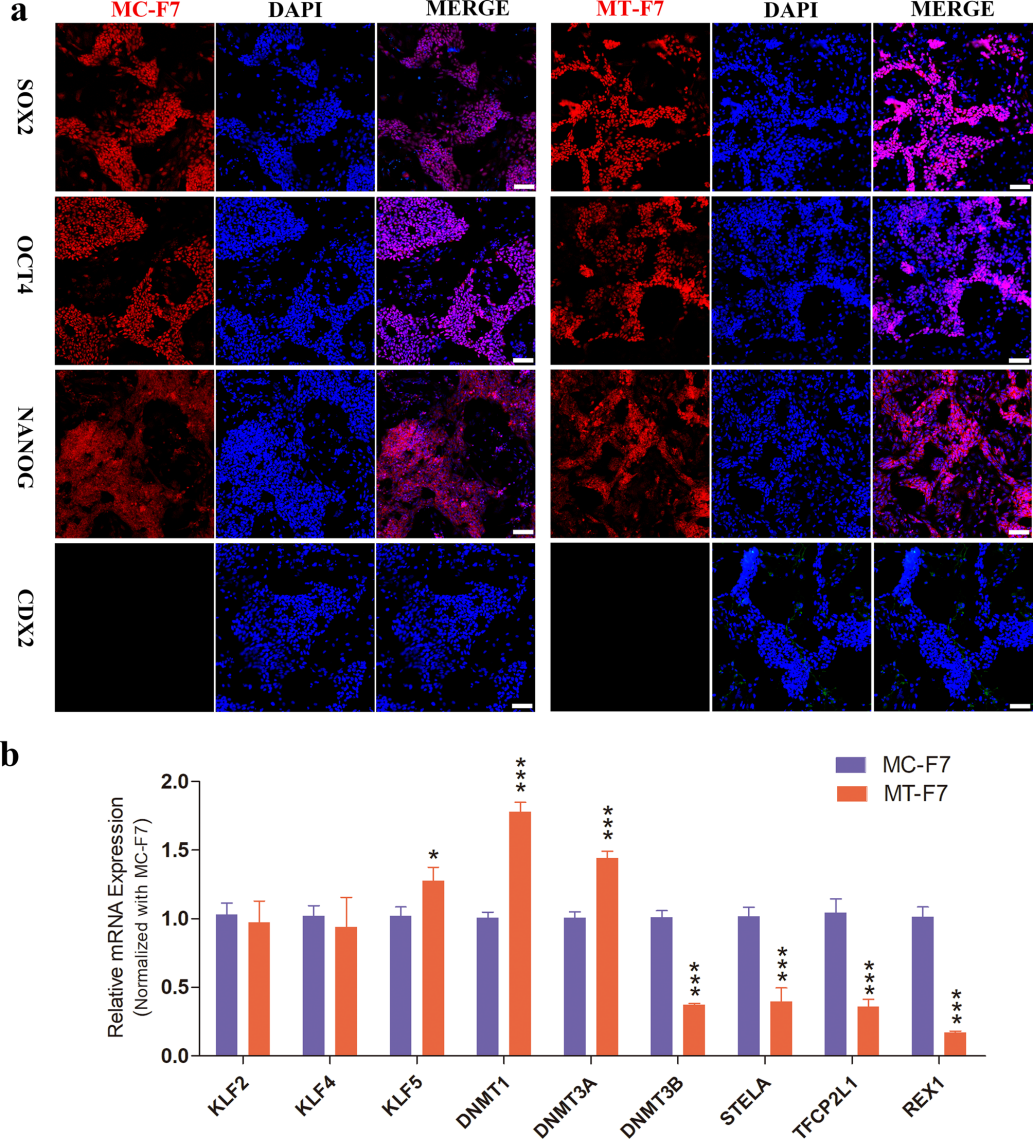
Immunofluorescence and RT-PCR analyses bESCs on methanol-fixed feeder layer. (**a**) Immunofluorescence staining images show the expression of SOX2, OCT4, NANOG, and CDX2 proteins in MC-F7 and MT-F7 cells. Bar = 100 μm. (**b**) RT-PCR analysis shows the expression levels of pluripotent markers such as KLF5, DNMT1, DNMT3A, DNMT3B, STELLA, TFCP2L1, and REX1 in the MC-F7 and MT-F7 cells.

RT-PCR analysis showed that the OCT4 and NANOG mRNA levels were slightly reduced in the MT-F7 cells compared to the MC-F7 cells (Fig. 1e). Western blotting and immunofluorescence staining analyses showed that MT-F7 cells are expressing most of the pluripotent regulatory factors such as OCT4, SOX2 and NANOG, but not CDX2 (Figs. 1f, 2a, Fig. S1a,aʹ). RT-PCR analysis showed that the expression levels of pluripotency regulatory genes such as KLF5, DNMT1 and DNMT3A were significantly increased in the MT-F7 cells compared to the MC-F7 cells, whereas the expression levels of DNMT3B, STELLA, TFCP2L1, and REX1 were significantly decreased in the MT-F7 cells (Fig. 2b).

**Figure 3:**
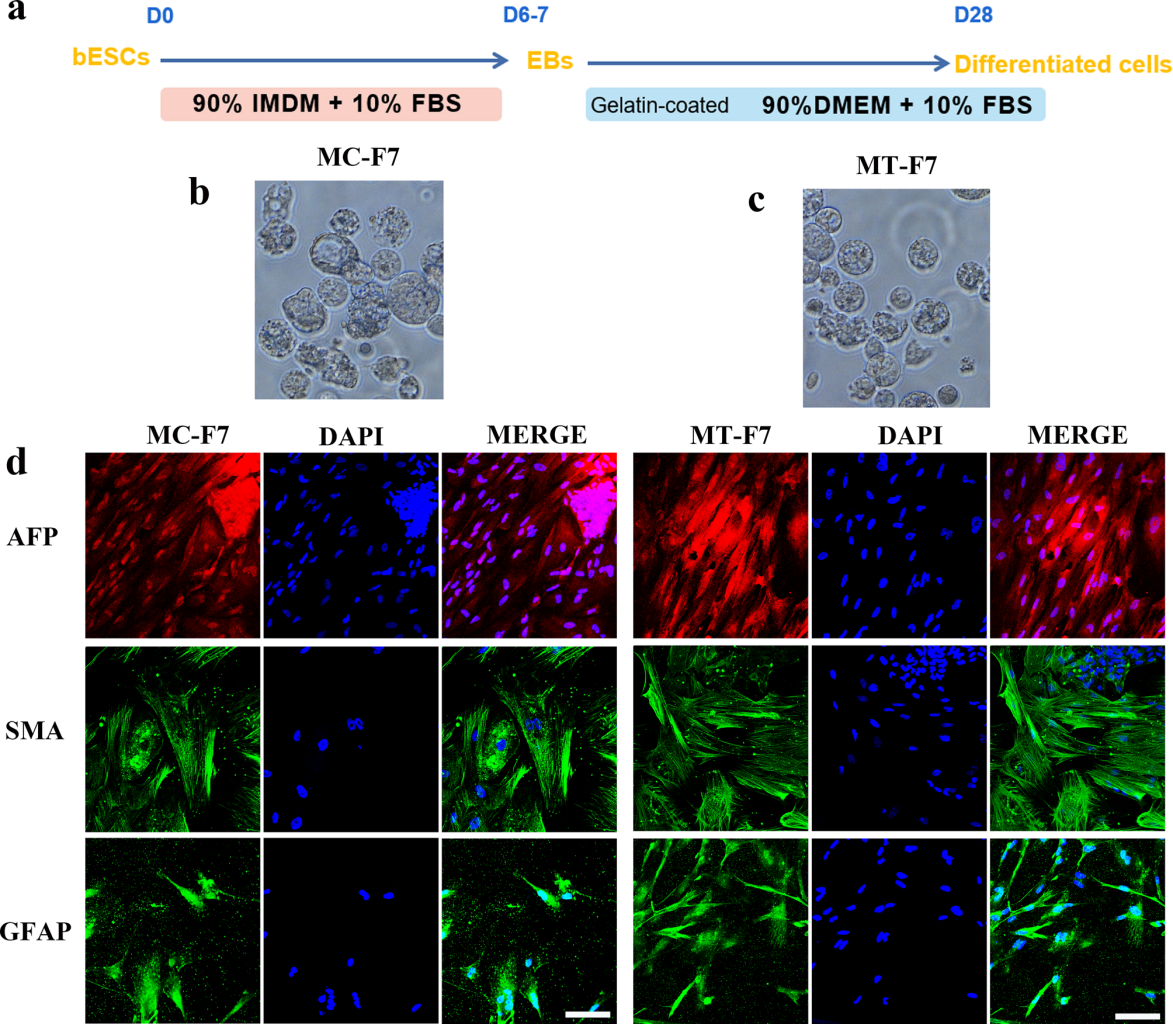
The bovine ESCs cultured on methanol-fixed MEFs show long-term differentiation potential. (**a**) Schematic diagram shows the experimental strategy for differentiation of bESCs. (**b,c**) The embryoid bodies (EBs) genrerated from MC-F7 and MT-F7 cells. (**d**) The differentiated MT-F7 and MC-F7 cells show high expression levels of endodermal (AFP), mesodermal (SMA), and ectodermal (GFAP) marker proteins. Bar = 100 μm.

The differentiation ability of the F7 grown on two different feeder layers (MC-MEFs and MT-MEFs) was tested by differentiating the bESCs at 23rd passage into embryoid bodies (EBs) (Fig. 3a–c). MT-F7 cells showed significantly higher expression of endodermal (AFP), mesodermal (SMA) and ectodermal (GFAP) marker proteins (Fig. 3d). This suggested that F7 grown on the chemically fixed feeders retained significant differentiation potential. In summary, long-term growth of bESC cells on the methanol-fixed feeder layer maintained pluripotency.

### The global expression profile of bESCs cultured on methanol fixed feeder layers and comparative analyses with bESCs cultured on mitomycin C treated feeder layers

**Figure 4:**
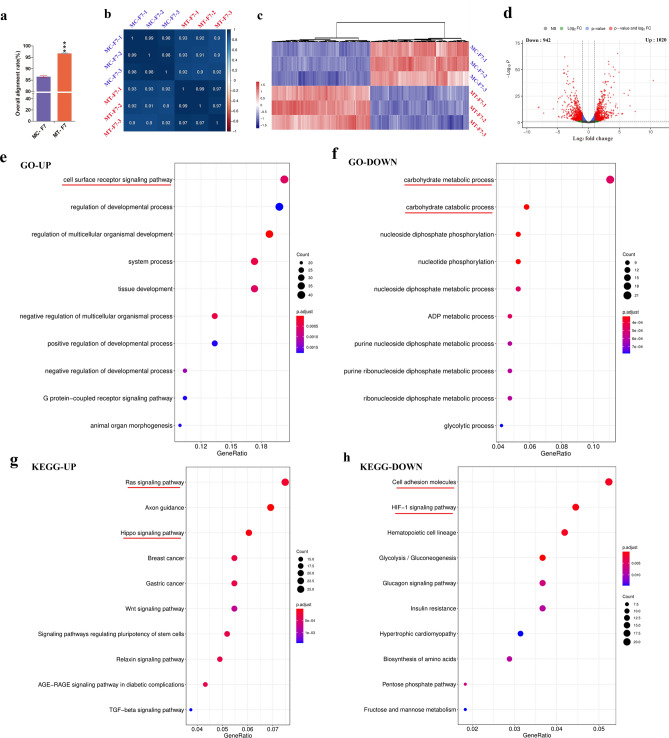
Global gene expression profile of MT-F7 and MC-F7 cells based on RNA-seq analysis. (**a**) The overall alignment rate of MT-F7 cells was greater than that of MC-F7 cells (96% vs. 85%). (**b**) Pearson correlation analysis showed similar global transcriptional profiles among samples of MC-F7 and MT-F7 cells. (**c**) Heatmap shows the fold changes of 1962 DEGs (|log2FC |> 1.5; P value < 0.05) in MT-F7 cells compared to the MC-F7 cells. The gene read counts were consistent between the MT-F7 and MC-F7 samples. (**d**) The volcano map shows significantly higher expression of 1020 genes and reduced expression of 942 genes in the MT-F7 cells compared to the MC-F7 cells. (**e–h**) The top 10 GO terms and KEGG pathways (up and down) based on the differentially expressed genes (DEGs) between MC-F7 and MT-F7 cells. The enriched GO terms or KEGG pathways with the most gene counts and Gene Ratio were analyzed at the genetic level (above the red lines).

RNA-seq was performed to compare the global RNA expression of bESCs grown on MC-MEFs and MT-MEFs. More than 85% of the readings of MC-F7 cells mapped to the reference genome (Bos taurus), thereby demonstrating that the RNA sequencing data was highly reliable and repeatable (Fig. 4a). Furthermore, the overall alignment rate of the MT-F7 cells was greater than 96% (Fig. 4a). This suggested that the growth on the chemically fixed feeder cells reduced feeder cell contaminations in the PSC cultures, which is very useful for single cell sequencing or other deep analyses. Pearson correlation analysis showed that the global transcriptional profile of the F7 grown on the two distinct kinds of feeder layers were similar (Fig. 4b). Heatmap and fold change values of the 1962 DEGs (|log2FC |> 1.5; P value < 0.05) showed that the gene read counts were consistent between the samples (Fig. 4c,d). The volcano map showed that 1962 genes were differentially expressed (increased expression of 1020 genes and decreased expression of 942 genes) in the MT-F7 cells compared to the MC-F7 cells (Fig. 4d). The top GO terms for upregulated DEGs of MT-F7 cells were the cell surface receptor signal pathway, cell development, organogenesis, and G protein coupled receptor signal pathway (Fig. 4e). The top GO terms for the downregulated DEGs included the carbohydrate metabolic process and carbohydrate catabolic process (Fig. 4f). The enriched KEGG pathways for the upregulated DEGs of MT-F7 cells were the Ras signaling pathway, Hippo signaling pathway, Wnt signaling pathway, signaling pathways regulating pluripotency of stem cells, Relaxin signaling pathway, and TGF beta signaling pathways (Fig. 4g). The enriched KEGG pathways for downregulated DEGs included cell adhesion molecules, HIF-1 signaling pathway, and glycolysis/gluconeogenesis (Fig. 4h).

Next, we verified the expression of 25 genes that related to pluripotency and the key catalytic enzymes of major metabolic pathways in bESCs by RT-PCR. The relative expression fold changes of these genes in RNA-seq and RT-PCR were compared. The results were consistent between these two assays for the up- or down-regulated genes, although fold changes were not exactly same (Fig. S2a). The correlation co-efficient between RNA-seq and RT-PCR results was 0.7976 (Fig. S2b). This confirmed the credibility of the RNA-seq data in this study.

RNA-seq analysis also showed that expression of some naïve-specific markers such as DPPA2, DUSP5, DUSP10, DNMT3L, CD9, ZEP42, and TFE3 were down-regulated in the MT-F7 cells compared to the MC-F7 cells. Whereas, the expression levels of DUSP3, DUSP14, STAT3, PRDM14, KLF5, HORMAD1, TET2, CD44, and MAEL were up-regulated in the MT-F7 cells (Fig. S3a). Furthermore, the expression levels of some primed-specific markers such as ZIC3, OTX2, DLL1, DNMT3A, NCAM1, RFX4, SOX6, MEIS2, LMO2, MEIS1, and ZNF521 were significantly higher in the MT-F7 cells compared to the MC-F7 cells (Fig. S3b). The expression levels of ESRRB, LEFTY2, SKIL, JARID2, and KAT6A were significantly reduced in the MT-F7 cells compared to the MC-F7 cells. Whereas, the expression levels of ZIC3, STAT3, HESX1, TCF3, FGF2, SMARCAD1, and SETDB1 were significantly higher in the MT-F7 cells (Fig. S3c). The expression levels of markers for the three germ layers did not show a global increase or decrease in the MT-F7 cells compared to the MC-F7 cells. The expression levels of ectodermal markers such as OTX1, RAX6 and NES as well as mesodermal markers such as TBX2 and KDR were significantly increased in the MT-F7 cells compared to the MC-F7 cells. Whereas, the expression levels of mesodermal markers such as NKX25, MYOD1 and EOMES as well as the endodermal markers such as GATA5 and FOXA1 were significantly decreased in the MT-F7 cells (Fig. S3d).

It is worth noting that the transcriptional level of genes encoding ECMs, such as collagen-IV, fibronectin and laminin, and growth factors-BMP4 and IGF2, were significantly higher in MT-F (except for TGF-β, Fig. S3e,f), indicating some MT-F7 differentiated into of feeder-like cells. In summary, bESCs grown on MT-MEFs showed unique gene expression patterns, but maintaining pluripotency compared to those grown on MC-MEFs.

### Functional enrichment analyses of the effects of methanol fixed feeder layers on the bESCs

**Figure 5:**
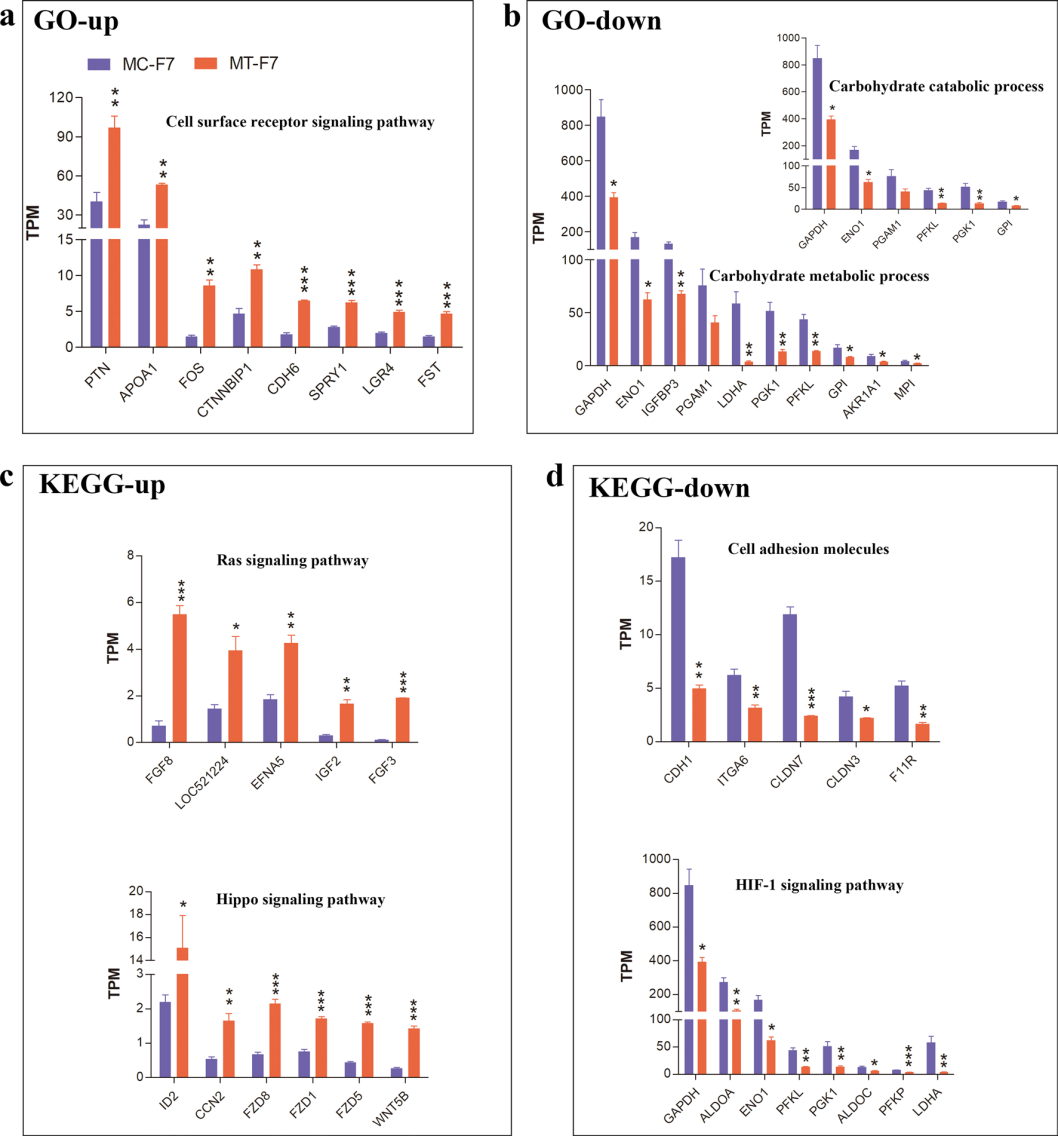
Functional enrichment analysis of differentially expressed genes in MT-F7 cells. The list shows top 5–10 genes with high transcript levels in each of the enriched GO terms or KEGG pathways based on the DEGs between MT-F7 and MC-F7 cells. (**a**) The cell surface receptor signaling pathway (GO up) is upregulated in the MT-F7 cells. (**b**) The carbohydrate metabolic process and carbohydrate catabolic process (GO down) is downregulated in the MT-F7 cells. (**c**) Ras and Hippo signaling pathways (KEGG up) are upregulated in the MT-F7 cells. (**d**) Cell adhesion genes (KEGG down) are downregulated in the MT-F7 cells.

To determine the effects of the methanol-fixed feeder layer on the bESCs, we further analyzed the enriched GO terms and KEGG pathways based on the DEGs with high gene counts and Gene Ratio (Figs. 4e–h, 5a–d). The mRNA expression levels of PTN and APOA1 in the cell surface receptor signaling pathway (up-regulated GO term) were significantly higher in the MT-F7 cells compared to the MC-F7 cells (Fig. 5a, Table S2), which suggested that MT-F7 cells expressed significantly higher levels of Pleiotrophin (a secreted growth factors that mediates its signal through cell-surface proteoglycan and non-proteoglycan receptors) and Apolipoprotein A-I (a cofactor for the lecithin cholesterol acyltransferase). However, the transcript levels of GAPDH, ENO1, IGFBP3, PGAM1, and LDHA genes, which are involved in the carbohydrate metabolism and catabolism were downregulated in the MT-F7 cells compared to the MC-F7 cells (Fig. 5b, Table S3), which suggested that carbohydrate metabolism and glycolytic pathway were down-regulated in the MT-F7 cells compared to the MC-F7 cells. Furthermore, KEGG pathway analyses showed that transcript levels of FGF8, IGF2, FGF3, ID2, CCN2, FZD1, and FZD8 in the Ras and Hippo signaling pathways were up-regulated in the MT-F7 cells compared to the MC-F7 cells (Fig. 5c, Table S2). This upregulation suggested increased expression of growth factors (ligands), transmembrane receptors, adhesion molecules, and transcriptional regulators in the MT-F7 cells. Moreover, GAPDH, PFKL, PGK1 and ENO1 genes were observed concurrently in the carbohydrate metabolic process (GO down), carbohydrate catabolic process (GO down), and HIF1 signaling pathway (KEGG down) (Table S4). This observation suggested that GAPDH, PFKL, PGK1 and ENO1 et al., the catalytic enzymes of glycolysis pathway, play distinct roles in multiple biological processes and signaling pathways. We also observed significant down-regulation of cell adhesion genes, such as CDH1, ITGA6, CLDN3, and CLDN7 in the MT-F7 cells (Fig. 5d, Table S3), which suggested that decreased expression of proteins related to cell adhesion and tight junctions on the cell membrane surface of MT-F7 cells. The downregulation of HIF1 signaling pathway and HIF1A and HIF3A, indicated that MT-F7 were exposed to a relatively oxygen-rich environment because of metabolic termination of the MT-MEFs (Fig. 5d, Fig. S4b).

### Characteristics of central carbon metabolism in bESCs grown on methanol-fixed feeder layers

**Figure 6:**
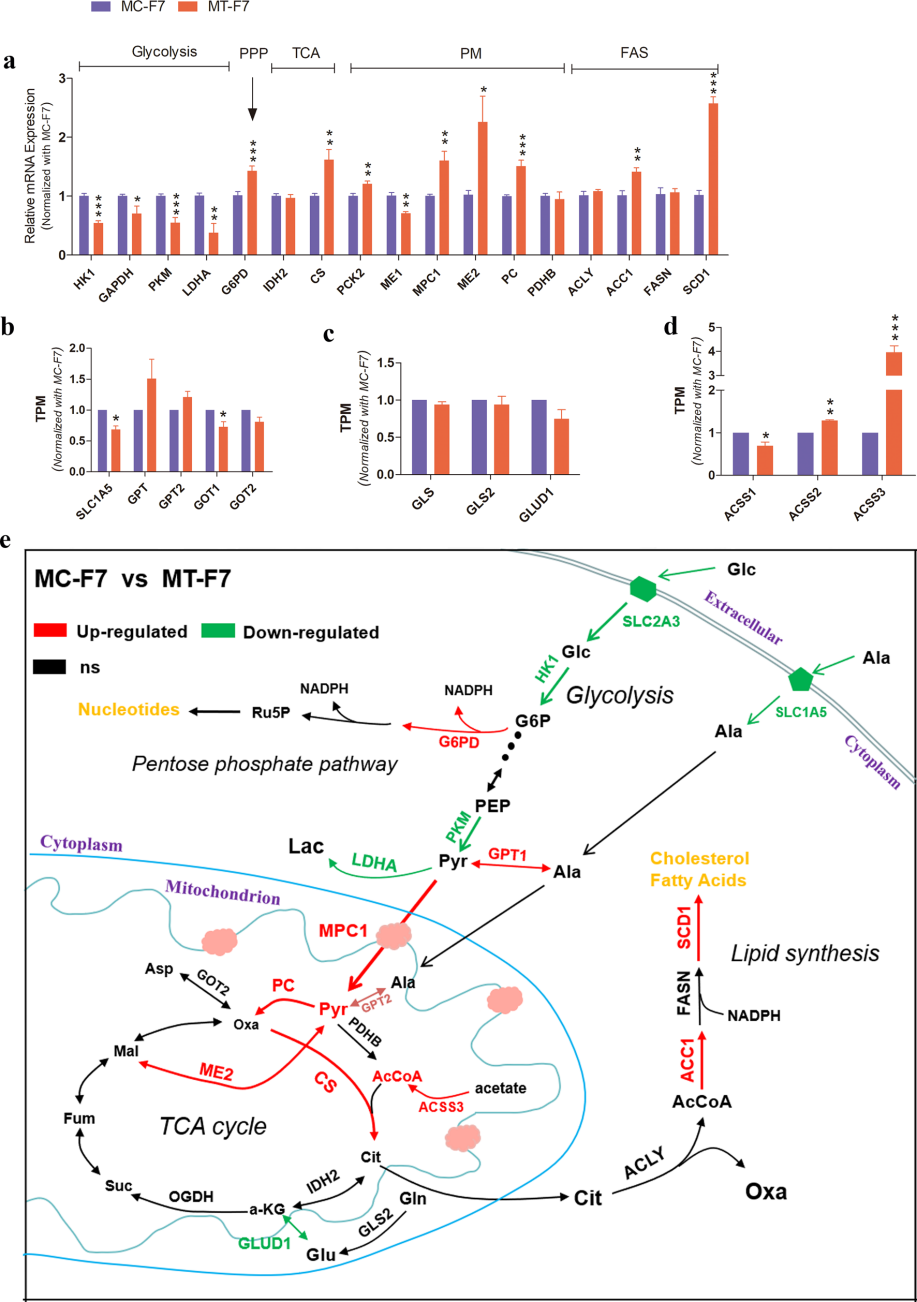
Characterization of central carbon metabolism in the F7 grown on MT-MEFs and MC-MEFs. (**a**) RT-PCR analysis showing the expression levels of the key catalytic enzymes, including rate-limiting enzymes in major metabolic pathways. *PPP* pentose phosphate pathway, *PM* pyruvate metabolism, *FAS* de novo fatty acid synthesis. (**b**) The relative expression of genes encoding catalytic enzymes related to pyruvate metabolism. (**c**) The relative expression of genes encoding catalytic enzymes related to glutaminolysis. (**d**) The relative expression of genes encoding catalytic enzymes related to acetyl CoA (AcCoA) biosynthesis (ACSS family). (**e**) The metabolic diagram shows the differential expression of genes mentioned above.

Glycolysis is very important for the pluripotency maintenance of PSCs. In the view of the DEGs enriched in glycolysis pathway, the metabolic status of the MT-F7 cells were different from MC-F7. The expression levels of SLC2A1, SLC2A3, SLC2A8, and SLC2A11 were significantly reduced and the expression levels of SLC2A5 and SLC2A6 were significantly increased in the MT-F7 cells compared to the MC-F7 cells (Fig. S4a). The transcript levels of glycolytic genes such as HK1, PKM, LDHA, and GAPDH were significantly reduced and the transcript levels of the gene encoding the pentose phosphate pathway (PPP) enzyme G6PD were significantly increased in the MT-F7 cells compared to the MC-F7 cells (Fig. 6a). The expression levels of IDH2 in the TCA cycle were not significantly different between the MT-F7 and MC-F7 cells, whereas the gene encoding citrate synthase (CS) was significantly increased in the MT-F7 cells compared to the MC-F7 cells (Fig. 6a). The expression levels of genes regulating pyruvate metabolism, such as, PCK2, MPC1, and PC were significantly higher in the MT-F7 cells compared to the MC-F7 cells (Fig. 6a). Furthermore, the transcript levels of ME1 and GOT1 were significantly decreased, and the expression of GPT was increased in the MT-F7 cells compared to the MC-F7 cells (Fig. 6a,b). The transcript levels of glutamine-metabolizing enzymes were comparable between the MT-F7 and MC-F7 cells (Fig. 6c). The transcript levels of genes encoding acetyl CoA (AcCoA) biosynthetic enzymes-ACSS2, ACSS3, and SCD1 were significantly higher in the MT-F7 cells compared to the MC-F7 cells (Fig. 6a,d). Interestingly, histone acetyltransferases KAT2A and KAT8 in histone acetyltransferase family were significantly increased in MT-F7 compared with MC-F7, while the other members were not significantly different (Fig. S4c). Figure 6e illustrates the expression differences of metabolic genes in various metabolic pathways.

### The methanol-fixed feeder layers maintains the pluripotency and alters the metabolism of bovine expanded PSCs

**Figure 7:**
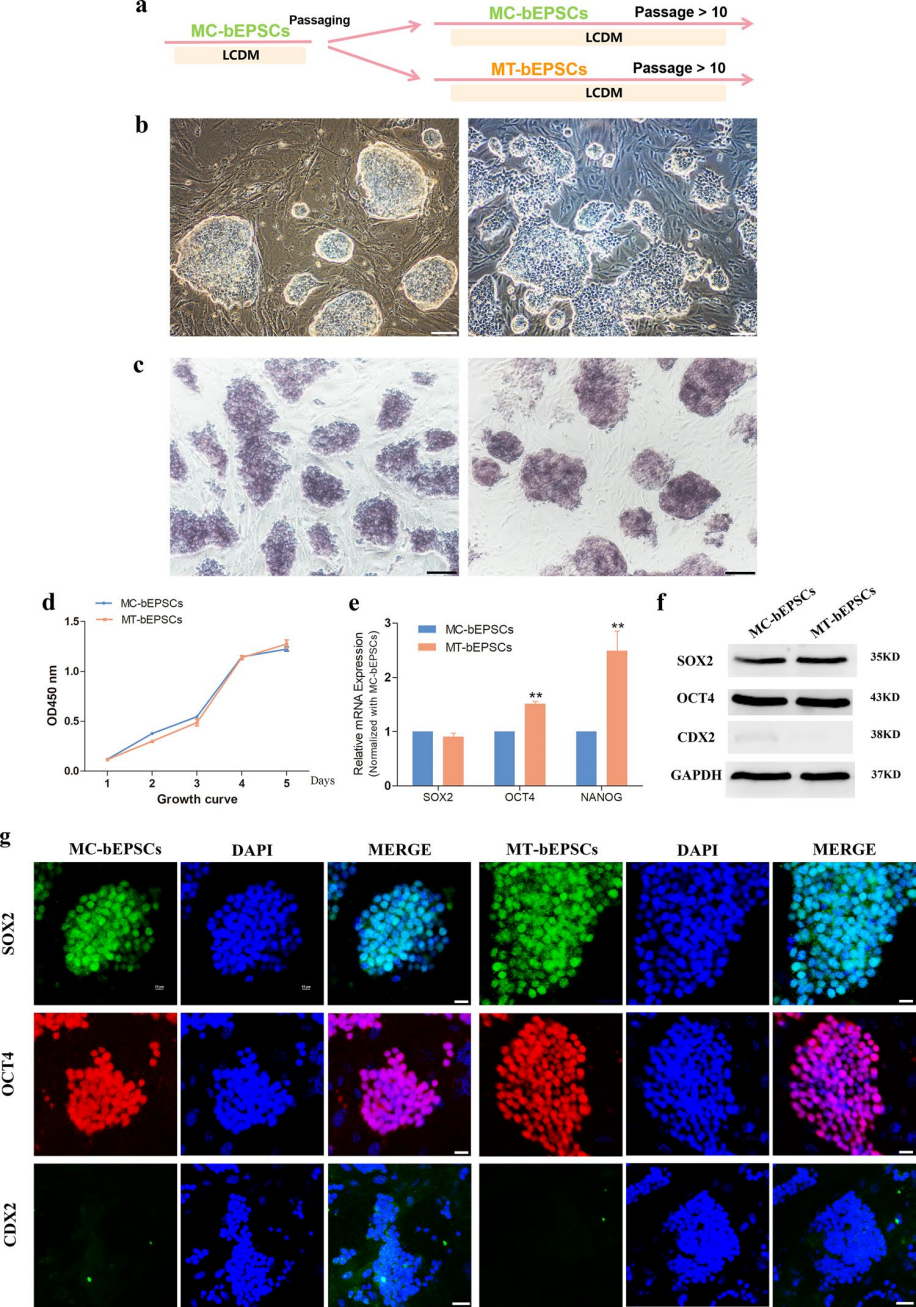
Effects of the methanol-fixed feeder layer on the bEPSCs. (**a**) The schematic diagram shows experimental strategy for analyzing bEPSCs grown on MT-MEFs and MC-MEFs. (**b**) The morphology of MC-bEPSCs and MT-bEPSCs. Bar = 100 μm. (**c**) AKP staining of MC-bEPSCs and MT-bEPSCs. Bar = 100 μm. (**d**) Growth curve of MC-bEPSCs and MT-bEPSCs. (**e**) RT-PCR analysis shows the expression of SOX2, OCT4 and NANOG in the MC-bEPSCs and MT-bEPSCs. (**f**) Western blotting analysis shows the expression levels of OCT4, SOX2, and CDX2 in the MC-bEPSCs and MT-bEPSCs. (**g**) Immunofluorescence staining assay results show the expression levels of SOX2, OCT4, and CDX2 in the MC-bEPSCs and MT-bEPSCs. Bar = 100 μm.

We then verified the reliability of the MT-MEFs as a feeder layer for bEPSCs. The bEPSCs were transferred from MC-MEFs to MT-MEFs during passaging, and the characteristic analyses were carried out after continuous culture for 10 passages (Fig. 7a). The colony formation, AKP staining, and the growth of bEPSCs on MT-MEFs and MC-MEFs were similar (Fig. 7b,d). The transcript levels of the core pluripotent regulators, OCT4 and NANOG, were significantly higher in the MT-bEPSCs compared to the MC-bEPSCs (Fig. 7e). Western blotting and immunofluorescence staining showed that the levels of SOX2 and OCT4 transcripts were comparable, and the CDX2 mRNA levels were not detected (Fig. 7f,g, Fig. S1b,bʹ).

**Figure 8:**
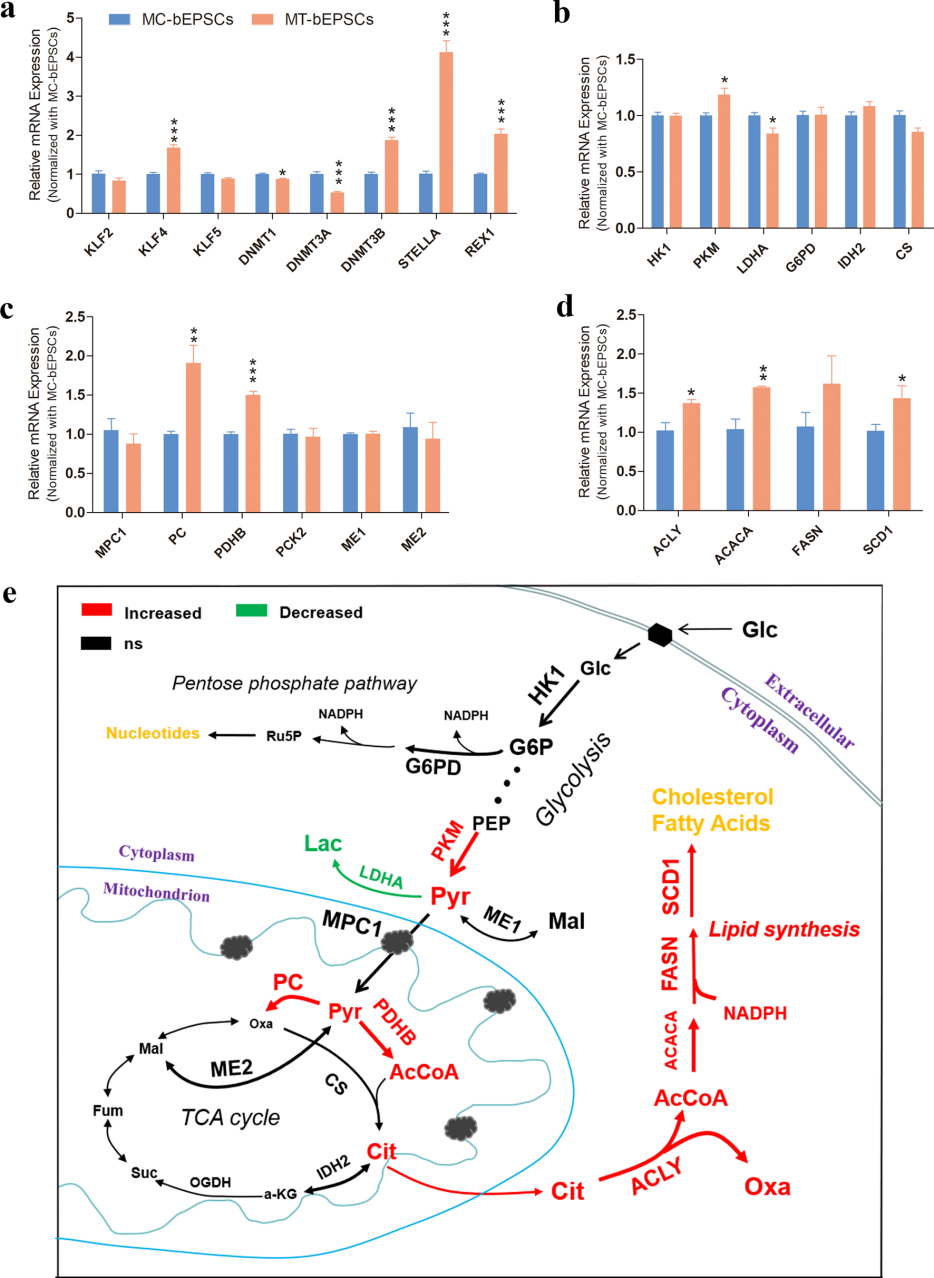
Characterization of central carbon metabolism in the bEPSCs grown on methanol-fixed MEFs. (**a**) RT-PCR analysis shows the expression levels of pluripotent regulatory genes, KLF2, KLF4, KLF5, DNMT1, DNMT3A, DNMT3B, STELLA and REX1, in the MC-bEPSCs and MT-bEPSCs. (**b**) RT-PCR analysis shows the expression levels of genes encoding key catalytic enzymes, HK1, PKM, LDHA, G6PD, IDH2 and CS, in major metabolic pathways. (**c**) RT-PCR analysis shows the expression levels of genes encoding the catalytic enzymes, MPC1, PC, PDHB, PCK2, ME1 and ME2, related to pyruvate metabolism. (**d**) RT-PCR analysis shows the expression levels of genes encoding the catalytic enzymes, ACLY, ACACA, FASN and SCD1, involved in de novo fatty acid biosynthesis. (**e**) The metabolic diagram shows differential expression of genes mentioned above.

Furthermore, RT-PCR results showed that the expression of the pluripotent related genes, such as KLF4, DNMT3B, STELLA, and REX1 and the key rate limiting enzyme of glycolysis, PKM, were significantly up-regulated in the MT-bEPSCs compared to the MC-bEPSCs (Fig. 8a,b). Moreover, the expression levels of pyruvate carboxylase (PC), pyruvate dehydrogenase B (PDHB), and genes encoding the catalytic enzymes related to fatty acid de novo biosynthesis were significantly higher in the MT-bEPSCs compared to the MC-bEPSCs (Fig. 8c,d). Figure 8e shows the differences in the expression levels of catalytic enzymes belonging to the main metabolic pathways in the MT-bEPSCs compared to the MC-bEPSCs.

## Discussion

The transmembrane ECMs and adhesion proteins that secreted by feeder cells retained after chemical fixation, which could maintain the pluripotency and differentiation potential of stem cells for a long time^13,15,29,37^. The chemical fixed feeder cells avoid the heterologous cell contaminations and provides inspiration for the application of artificial microenvironments in feeder-free culture. However, the previous studies did not show the global profiles in gene expression and the metabolic characteristics of stem cells grown on chemical fixed feeder layers^13,15,29,37^. In this study, MEFs were treated with methanol to generated chemical fixed feeder cells. The pluripotency and metabolism of bESCs cultured on MT-MEFs were compared with MC-MEFs from the perspective of global gene expression profiles.

Our results showed that methanol fixed MEFs could maintain the self-renewal and differentiation potential of bESCs over 20 passages, but generated the different molecular expression patterns and metabolic alteration for bESCs. The DEGs enriched in GO terms and KEGG pathway used to analyze the transcriptome sequencing data, and the function of the proteins encoded by DEGs was annotated according to the Uniprot database (https://www.uniprot.org/). We presumed the DEGs enriched in the GO terms and KEGG pathways possessing the largest number of genes and relatively significant difference generally represent the main molecular expression differences between MC-F7 and MT-F7.

Cell surface receptor signaling pathway (CSR) is a series of molecular signals originated by activation of the receptors on the surface of cells, and finales with regulation of downstream cellular processes, e.g. transcription. In this study, we found that the up-regulation of genes in CSR involved in early embryonic development and stem cell characteristics (Fig. 5a, Table S2), which indicated bESCs cultured on MT-MEFs still exhibited the characteristics of PSCs. While the up-regulation expression of EFNA5, FGF8 and FGF3 enriched in the RAS signaling pathway indicating some differentiations of bESCs grown on the MT-MEFs. The Ras proteins are GTPases that function as molecular switches for signaling pathways regulated cell proliferation, survival, growth, migration and differentiation. Activated RAS (RAS-GTP) regulates multiple cellular functions through effectors including Raf, phosphatidylinositol 3-kinase (PI3K), and Ral guanine nucleotide-dissociation stimulator (RALGDS)^38–41^. EFNA5 is the Eph receptor on the cell surface, a family of receptor tyrosine kinases, which plays an important role in migration and adhesion during the development of neurons, blood vessels and epithelium^42,43^. FGF3 and FGF8 are involved in the regulation of embryonic development, proliferation, differentiation and migration, and are required for normal brain, eyes, ears and limb development during embryogenesis^44–48^. However, the increased expression of ECMs and growth factors might display the self-adaptive ability of bESCs to the change of feeder cells. The previous studies have shown that the IGF-II/IGF1R axis plays a central and direct role in the self-renewal and pluripotency maintenance of human embryonic stem cells (hESCs). Blocking the IGF-II/IGF1R pathway will reduce the survival rate and clone formation ability of hESCs^49^. Here, the increased expression of IGF2 gene might reveal that bESCs antagonizes differentiation through self-regulation. The Hippo signaling, a highly conserved signaling pathway from insects to mammals regulates organ size and participates in the normal development of the embryo^50–53^. The upregulation of genes enriched in this pathway reveals that some differentiated cells may generate from bESCs.

The cell adhesion molecules (CAMs) are involved in cellular adhesion within the microenvironment; their interaction with the surface ligands leads to the activation of crucial signaling associated with the promotion of stemness and self-renewal capacity^54,55^. As a member of the typical cadherin families, CDH1 (E-cadherin) is essential in epithelialization, cell rearrangement, histogenesis, establishment of cell polarity and maintenance of tissue structure in early mouse embryos^56^. E-cadherin represents a marker for undifferentiated cells; the high expression of E-cadherin correlated with the promotion of the pluripotency and self-renewal potential of hESCs^57,58^. E-cadherin-mediated cell–cell adhesion is critical for the pluripotency and the dense clonal morphology of the mESCs in the in vitro cultures^59,60^. E-cadherin gene knockout (Cdh1^−/−^) leads to the loss of the ability of compaction during early embryonic development^61^. Therefore, the flattened phenotype of MT-F7 clones may attribute to the decreased levels of CDH1, CLDN3 and CLDN7 enriched in CAMs.

The microenvironment must retain low levels of oxygen in order to minimize the damage caused by DNA oxidation in stem cells^62^, and hESCs cultured under hypoxic conditions show lower spontaneous differentiation. However, this state could reverse by inhibiting the Notch pathway or transferring the culture to normal conditions^63,64^. Under hypoxic conditions, the activation of HIF1 promotes the transcription of glucose transporter and glycolytic enzyme genes, which plays a key role in the maintenance of glycolysis phenotype^65–70^. Glycolysis is the central pathway of glucose metabolism. The absorption and exit-flux of glucose must be balanced, and the flux through glycolysis must maintained by the enzymes in glycolysis^71^. In this study, the expression of HIF-1 genes decreased relatively in MT-F7, and the genes enriched in the down-regulated HIF1 signaling pathway, carbohydrate metabolic process and carbohydrate catabolic process (except for MPI, GPI, IGFBP3 and AKR1A1 in Fig. 5b,d), belong to the main catalytic enzymes in the glycolysis pathway. The decreased glycolysis in MT-F7 would lead to the reduction of pyruvate and pyruvate-AcCoA flux, and the extremely low density of exogenous fatty acids in CTFR could not support the fatty acids oxidation to produce AcCoA. Pyruvate, junction point of glycolysis and TCA cycle, was the key intermediate of the glycolysis pathway, and the upstream substrate of lactate (Lac). Wellen et al. proposed a new mechanism to link glucose metabolism to chromatin modification and overall transcriptional control through ATP-citrate lysate and the production of acetyl-CoA (Acetyl-CoA)^72^. And Arieh Moussaieff et al. have proved that the Pyruvate-AcCoA pathway plays an important role in maintaining the pluripotency of ESCs by increasing histone acetylation^73^. Here, the decreased LDHA expression in MT-F7 might relate to the decrease of pyruvate-lactate flux. This may lead to the main flux of pyruvate producing more AcCoA through the pyruvate-AcCoA pathway. The increased expression of MPC1 indicates transfer pyruvate into the mitochondria for AcCoA production^74^. The increased expression of GPT1 and GPT2 could catalyze more alanine converting to pyruvate. In addition, the up-regulated ACSS2 and ACSS3 might catalyze more acetate to AcCoA^75^. Increased expression of KAT2/5/8 might play a positive role in the maintenance of histone acetylation in MT-F7 (Fig. S4c). Wang et al. have found that fatty acid (FA) synthesis activation is critical for stem cell pluripotency^33^. Our results show that the expression level of FA synthases in MT-F7 was maintained or increased. The reduction of glycolysis was disadvantageous to the maintenance of bESCs pluripotency; however, the adjustment of metabolic patterns to make up for this deficiency might be advantage to the maintenance of bESCs identity.

The differences of the global transcript expression and metabolism between MT-F7 and MC-F7 mainly due to the fixed MEF lose metabolic, as well as the ability of secretion of growth factors and other cytokines. This led to the shortage of growth factors derived from MEFs. The previous studies have shown that MEF produces factors needed for hESC pluripotency, including known signaling receptor ligands, extracellular proteases and extracellular matrix components, which might relate to the signaling transduction of PSCs^76^. The FGF2 treatment on MEF is essential for the production of these factors, and in the absence of FGF2, these secretory factors are down-regulated^76^. Moreover, the quantitative and semiquantitative immunoassay of growth factors and cytokines in the conditioned medium of STO and CF-1 mouse feeder cells show that the most abundant cytokine proteins expressed in MEF were Activin A, hepatocyte growth factor (HGF), insulin-like growth factor 1 (IGF1) and insulin-like growth factor 2 (IGF2)^11^. And Eiselleova et al. confirmed that the presence of FGF2 could increase the secretion of TGF-βand Activin A from MEFs, while MEF itself hardly secretes FGF2^9^. Here, however, the interruption of MEF-derived growth factors did not lead to complete differentiation of bESCs grown on MT-MEFs. Not only that, bESCs showed heterogeneous expression of pluripotent markers, and possessed the identical differentiation ability after 23 passages. Bendall et al. have demonstrated that the self-renewal and pluripotent properties of hESCs, grown on Matrigel with condition medium, depend on the dynamic interaction between hESCs and autologously hESCs derived fibroblast-like cells (hdFS), while inhibition of the FGF pathway indirectly leads to hESCs differentiation^49^. Similarly, bESCs grown on the MT-MEFs increased the expression of ECMs and growth factor genes, indicated that some bESCs derived fibroblast-like cells might make up for the lack of secretion from feeder layers. Hence, we speculate that the alteration of glucose metabolism, including the decline of glycolysis, seems to relate to the lack of some known signaling receptor ligands in the culture medium, such as Activin A or IGF2, which might relate to signaling transductions.

The overall metabolic characteristics, especially the down-regulation of the glycolysis pathway and the up-regulation of development-related genes, suggest that the MT-MEFs could not completely replicate the state of bESCs on the traditional feeder layer; in other words, the culture system might requires the addition of some growth factors and the up-regulation of signal pathways related to carbohydrate metabolism. Our study provide a reference for the optimization of the culturing system of bESCs, which included supplementation of growth factors (such as Activin A, IGF2, TGF-β, etc.), up-regulation of the glycolysis pathway (agonists of glycolysis metabolic enzymes), inhibition of differentiation-related signaling pathways, and maintenance of hypoxia environment.

Significantly, the bEPSCs grown on methanol-treated MEFs exhibited up-regulation of the three core pluripotency regulators and some important pluripotent genes. Metabolically, the expression levels of key glycolytic rate-limiting enzymes and fatty acid de novo synthases coding genes were higher in MT-bEPSCs. The activity of important metabolic pathways such as glycolysis and fatty acid de novo biosynthesis are positively involved with the pluripotency maintenance of stem cell^31–33^.

According to the results of this study, MT-MEFs seem to have limitations in maintaining pluripotency of bESCs, but their applicability seems to be different depending on the culture conditions or type of stem cells. We speculate that methanol-fixed MEFs may be more suitable for the growth of naïve-state embryonic stem cells than primed-state stem cells, due to the mESCs^13^ exhibited a similar phenomenon to bEPCs on MT-MEFs. In addition, the adjustment of medium conditions is closely related to the maintenance or transformation of the pluripotent state of stem cells^77^, but the specific mechanism is still unsolved.

## Conclusions

The methanol-fixed MEFs were efficient feeder layer, which can maintain the self-renewal of bPSCs. While the pluripotent markers and the metabolic characteristics of the bPSCs were altered this feeder layer.

## Supplementary Information

Supplementary Information.

## Data Availability

All data generated or analyzed during this study are included in this published article and its Supplementary Information files. Gene expression data of MC-F7 and MT-F7 are available in the GEO databases under the accession number GSE199731.
