# Supplementary material for: Methanol fixed feeder layers altered the pluripotency and metabolism of bovine pluripotent stem cells
Source: Sci Rep. 2022 Jun 2;12:9177. doi: 10.1038/s41598-022-13249-3 (PMC9163156; doi:10.1038/s41598-022-13249-3)
Supplement: Supplementary file 1 — Supplementary Information. [file 41598_2022_13249_MOESM1_ESM.docx]

**Supplemental Information**

**Methanol fixed feeder layers altered the pluripotency and metabolism of bovine pluripotent stem cells**

Wenqiang Xu[^1^](#第1单位)^,2^, Ruifeng Hao[^1^](#第1单位), Jing Wang[^1^](#第1单位), Lingna Gao[^1^](#第1单位), Xuejie Han[^1^](#第1单位), Chen Li[^1^](#第1单位), Shu Fang[^1^](#第1单位), Hui Zhang^3^, Xueling Li ^1^[^*^](#OLE_LINK2)

^
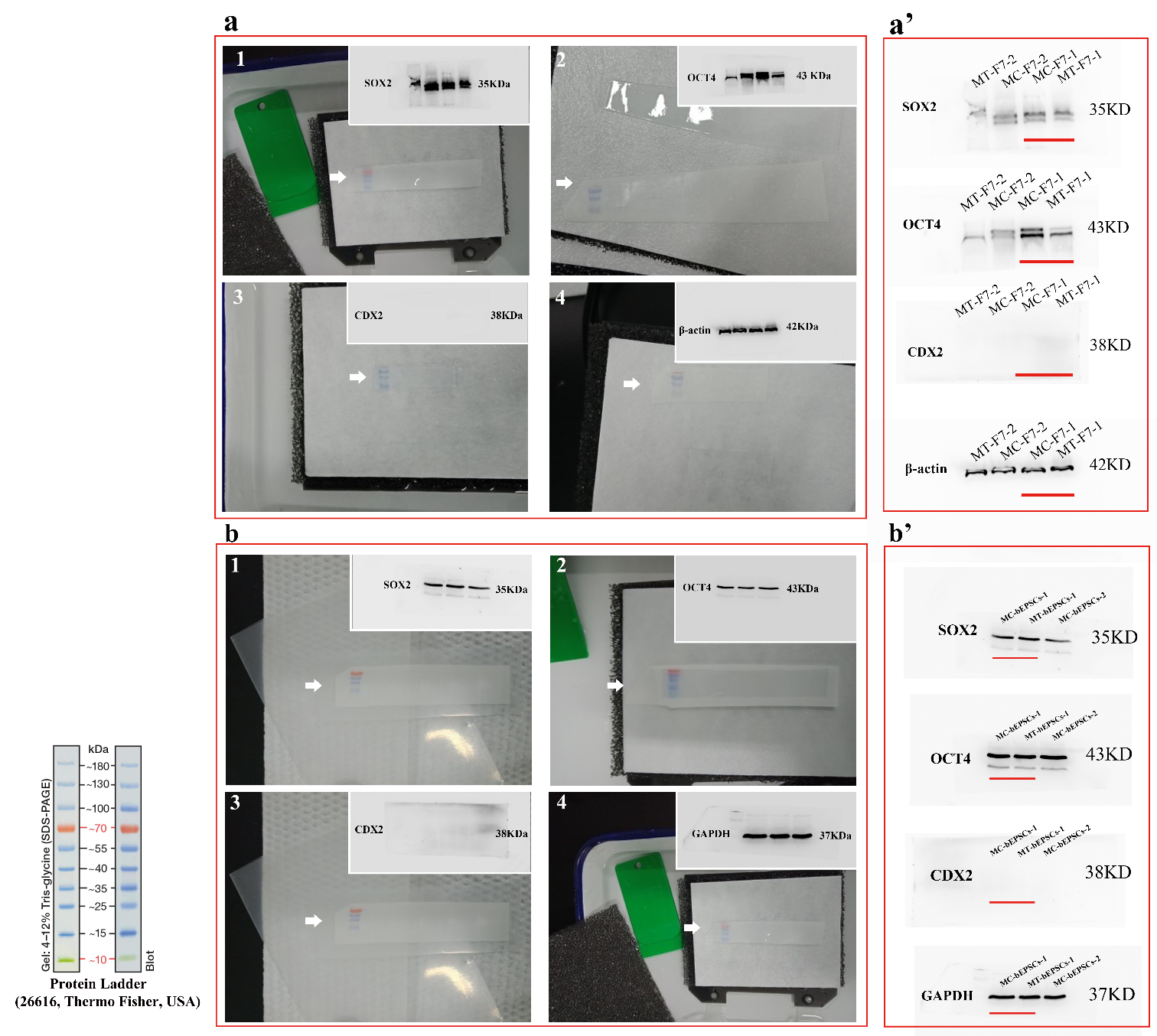
^

**Figure S1 The full-length membranes and cropped blots for Western botting.** (a, a’) The full-length membranes and cropped blot of SOX2, OCT4, CDX2 and ß-actin of bESCs cultured on MT-MEFs and MC-MEFs. (b, b’) The full-length membranes and cropped blot of SOX2, OCT4, CDX2 and GAPDH of bEPSCs cultured on MT-MEFs and MC-MEFs. The transferred membranes was indicated by arrows, which is located at the left side of each membrane in each image containing the protein ladder produced by Thermo Fisher with the red band represents 70 KDa. The developed blots in the upper right corner of each image showing the bands of the internal reference proteins and the target proteins, but exclude the bands of the protein ladders.


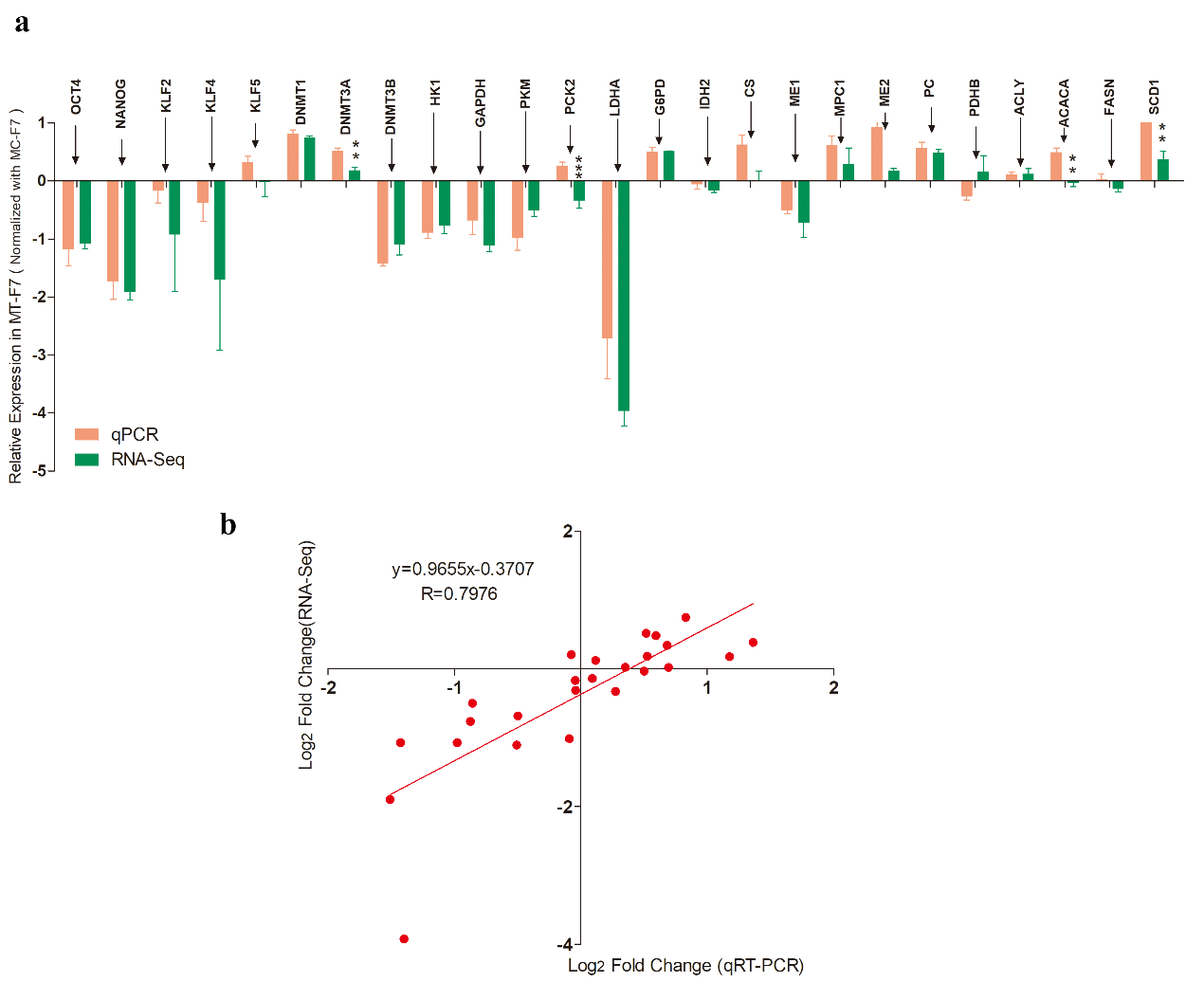


**Figure S2 Validation of the availability of RNA-seq by RT-PCR.** (a) The genes related to pluripotency regulators and key catalytic enzymes in major metabolic pathways of stem cells were selected for the validation of the availability of RNA-seq by RT-PCR. (b) The accordance of RNA-seq and RT-PCR were confirmed by a correlation coefficient of 0.7976, further indicated the credibility of RNA-seq in this study.

**
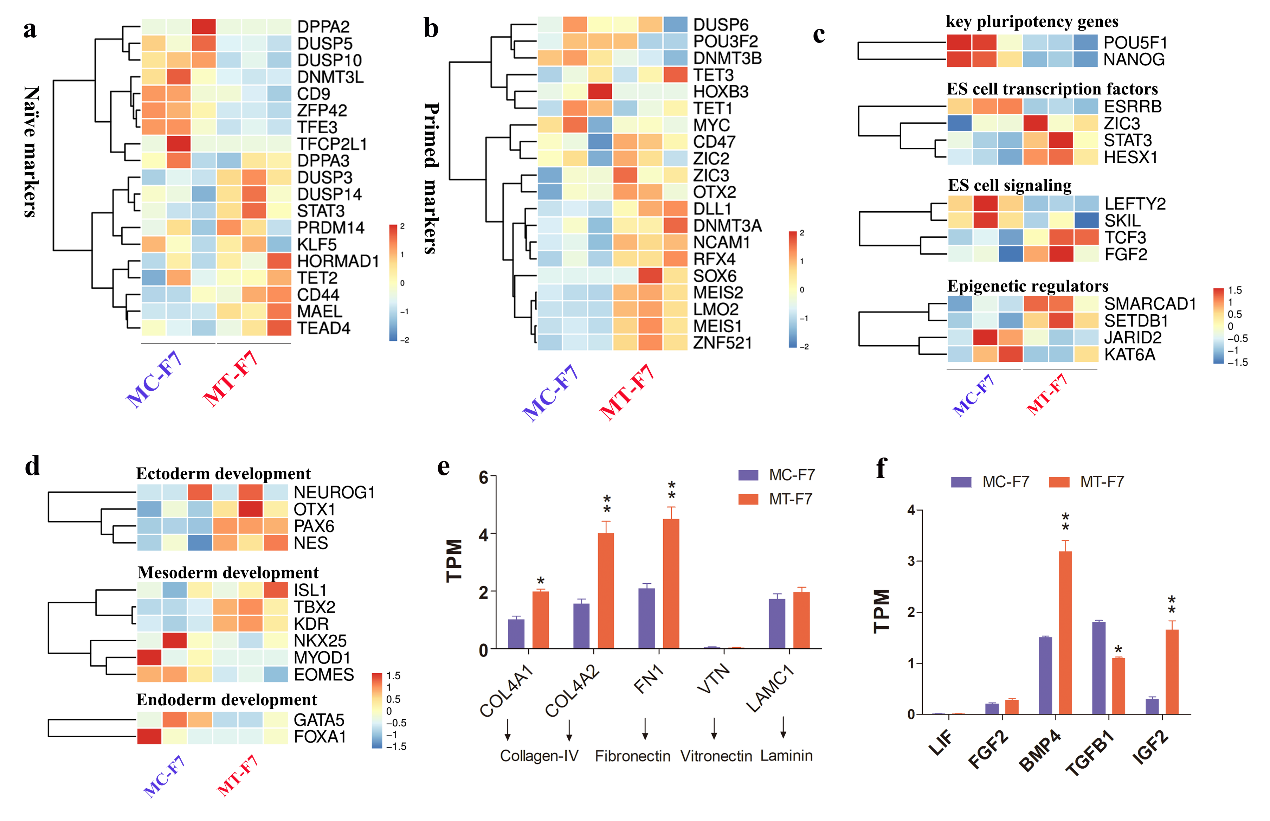
**

**Figure S3 Effects of methanol-fixed feeder layer on the expression of pluripotent factors and differentiated marker of bESCs (RNA-seq)**

(a, b) The differences of naïve and primed marker genes in MC-F7 and MT-F7. (c) The differences of pluripotent factors in MC-F7 and MT-F7. (d) The differences of three germ layers marker genes in MC-F7 and MT-F7.(e) The differences of extracellular matrix protein coding genes in MC-F7 and MT-F7. (f)The differences of growth factor coding genes in MC-F7 and MT-F7.


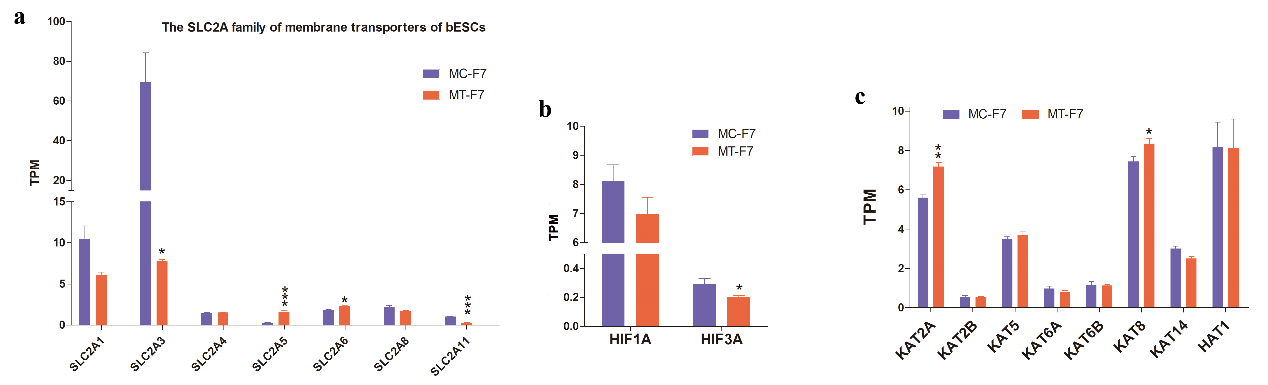


**Figure S4** (a) The differences of SLC2A family in MC-F7 and MT-F7. Among the members of SLC2A family, SLC2A1 and SLC2A3 showed the highest transcriptional levels. (b) Differences of gene expression of HIF1A and HIF3A in MC-F7 and MT-F7. (c) Differences of gene expression of KATs in MC-F7 and MT-F7.

**Supplemental Procedure**

***Bovine extended pluripotent stem cells (bEPSCs) culture***

Bovine EPSCs were provided by Inner Mongolia University. The LCDM medium prepared as previous report [1], which contains equal volume of DMEM/F12 (Thermo Fisher Scientific, USA) and Neurobasal (Thermo Fisher Scientific, USA), and supplemented with 0.5% N2 supplement (Thermo Fisher Scientific, USA), 1% B27 supplement (Thermo Fisher Scientific, USA), 1% nonessential amino acids, 1% L-glutamine, penicillin/streptomycin, 0.1 mM b-mercaptoethanol, 5% knockout serum replacement (Thermo Fisher Scientific, USA), 10 ng/mL recombinant human LIF (L, Millipore, USA), 2 mM (S)-(+)-dimethindene maleate (D, Tocris, UK), 1 mM CHIR99021(C, Tocris, UK), and 2 mM minocycline hydrochloride (M, MCE, USA).

bEPSCs were co-cultured with MC-MEF or MT-MEF at 37℃and 5% CO_2_, and the culture medium were refreshed every 24 hours. When reached about 80% confluence, the cells were dissociated by TrypLE (Gibco, USA) and platedon to the newly prepared MC-MEF or MT-MEF. The MC-bEPSCs were purified by velocity dependent adhesion method mentioned above before collection, while the MT-bEPSCs could be collected directly by centrifugation.

***Growth curve-bEPSCs***

The CCK-8 cell count kit (Solarbio, China) was used to test the growth rate of bEPSCs. In short, 3.6x10^3^ stem cells were plated on the resuscitated MC-MEF or MT-MEF during passaging (48-well plate). The MC-MEFs or MT-MEFs immersed in the LCDM medium (stem cells free) were used as parallel blank control to correct the OD value. 20 μl of CCK-8 reagent was added to each well and incubated at 37 °C and 5% CO2 for 3h. The absorbance OD values at 450 nm were measured every 24 hours for 5 days. The culture medium in the remaining well were refreshed after tests.

***Other methods are the same as for bESCs***

**References**

Yang Y, Liu B, Xu J, Wang J, Wu J, Shi C, Xu Y,Dong J, Wang C, Lai W et al. (2017) Derivation of pluripotent stem cells with in vivo embryonic and extraembryonic potency. Cell 169, 243–257, e25.

**Table S1 Primers for RT-PCR (*Bos taurus*)**

| Gene | Forward primer (5'->3') | Reverse primer (5'->3') |
| --- | --- | --- |
| *OCT4* | GGTTCTCTTTGGAAAGGTGTT | ACACTCGGACCACGTCTTTC |
| *SOX2* | CATCCACAGCAAATGACAGC | TTTCTGCAAAGCTCCTACCG |
| *NANOG* | TTCCCTCCTCCATGGATCTG | ATTTGCTGGAGACTGAGGTA |
| *CDX2* | CTCCTGGACAAGGACGTGAG | ACATGGTATCCGCCGTAGTC |
| *GAPDH* | GGGTCATCATCTCTGCACCT | GGTCATAAGTCCCTCCACGA |
| *DNMT3L* | ATGAGCAACTGGGTCTGCTT | GGGCTCTCTCTTCCACACAG |
| *DNMT3A* | CTGGTGCTGAAGGACTTGGGC | CAGAAGAAGGGGCGGTCATC |
| *DNMT3B* | CCGCAGATCAAGCTCAC | GTTATTTCGGGTTCGGAC |
| *DNMT1* | AGTGGGGGACTGTGTTTCTG | TGTACGAGAGCTGCATGTCC |
| *PRDM14* | CGGAGACAATTCCCTGATGT | CACGGGAATGTCCAGAAACT |
| *KLF2* | CCCTTCCAAACTGTGACTGG | GAGGATCGTGGTCTTCTCCC |
| *KLF4* | TCCCACCGCTCCATTAC | ATGAGAACTCTTCGTGTAGG |
| *KLF5* | TGTGCTTGCAGTGTTCTTCC | TGAATTGCCAGGTATGCTGC |
| *HK1* | CCTGATGGGACTGAGAACGG | ATTTCAATGGGAATGGCGTA |
| *PKM* | CACGAGTACCACGCAGAGACCA | TGCCCTTGATGAGCCCAGTTC |
| *LDHA* | AGTGTGCCTGTATGGAGTGGAG | TAATCATGGTGGAAATCGGATG |
| *G6PD* | TGAGGGTCGTGGGGGCTACTTT | GCTCGTTGCGCTTGCACTGCTG |
| *CS* | CCCTGACAGATCCCCGAGTT | GGCAGCAGTTTCTGGCATTC |
| *IDH2* | ATGGACGGCGATGAGATGAC | CCATTGGGGCTCTTCCACAT |
| *FASN* | TGCCTCCCTCAACTTCAAAAGA | CAAGTGGGAAAACACGGCAG |
| *MPC1* | TTTGCCCTCTGCTGTTACTCC | AGCCTTTTTAGACATCTCGTG |
| *ME2* | AGGACTTCTACCTCCCAAAAT | TCTCTCCATCAGTCACCACGA |
| *ME1* | TCTTGGTGTTGTGGCGTGTGG | AATGCTTCCTTGTTTGGGGGT |
| *PDHB* | TTCAGTCTGTGCCCATAGTCTT | CGTGTGTTCCTTGCCTTTCTAT |
| *PC* | GTTTCAGAGACATCCTGCTACG | CGCAGAACTTGAAGACCACATT |
| *PCK2* | CCCATCGGACTGGTGCCTAA | ATCCTGGTTGACCTGTTCTGT |
| *REX1* | GGAAGAGGACCCACTCCTTC | ACTTGGCCTCCTAGTGCATC |
| *ACTB* | GCGGCATTCACGAAACTACCTT | TCCTGCTTGCTGATCCACATCT |
| *HMBS* | TATTCGGGGAAACCTCAACA | CCCTGACCCACAGCATACAT |

**Table S2 The main function of the proteins encoded by the concerned genes.** The red font indicates the genes with relative increased level of expression in MT-F7 (MC-F7 as control). The annotations from uniprot (<https://www.uniprot.org/>) exhibited the main functions of proteins in Bos taurus species.

| **Gene ID** | **Gene names** | **Protein names(** from Uniprot : https://www.uniprot.org/**)** | **Main function(** from Uniprot : https://www.uniprot.org/**)** | **Entry** |
| --- | --- | --- | --- | --- |
| 280904 | **PTN** | Pleiotrophin (PTN) (Heparin-binding brain mitogen) (HBBM) (Heparin-binding growth factor 8) (HBGF-8) (Heparin-binding growth-associated molecule) (HB-GAM) (Heparin-binding neurite-promoting factor) (p18) | Secreted growth factors, participate in signal transduction and regulate cell proliferation, survival, growth, differentiation and migration. | [P21782](https://www.uniprot.org/uniprot/P21782) |
| 281631 | **APOA1** | Apolipoprotein A-I (Apo-AI) (ApoA-I) (Apolipoprotein A1) [Cleaved into: Proapolipoprotein A-I (ProapoA-I); Truncated apolipoprotein A-I] | Cofactors, involved in the binding and transfer of lipids. | [P15497](https://www.uniprot.org/uniprot/P15497) |
| 280795 | **FOS** | Proto-oncogene c-Fos (Cellular oncogene fos) | Nuclear phosphoprotein,plays an important role in TGF- β signal transduction, cell proliferation and differentiation. | [O77628](https://www.uniprot.org/uniprot/O77628) |
| 614630 | **CTNNBIP1** | Beta-catenin-interacting protein 1 | Interacting protein, Prevents the interaction between CTNNB1 and TCF family members, and acts as negative regulator of the Wnt signaling pathway. | [Q5E9N2](https://www.uniprot.org/uniprot/Q5E9N2) |
| 537946 | **CDH6** | Cadherin-6 | Calcium-dependent cell adhesion proteins. Interact with themselves in a homophilic manner in connecting cells;Contribute to the sorting of heterogeneous cell types. | [Q3SWX5](https://www.uniprot.org/uniprot/Q3SWX5) |
| 507095 | **SPRY1** | Protein sprouty homolog 1 (Spry-1) | Protein sprouty homolog, Inhibits fibroblast growth factor (FGF)-induced retinal lens fiber differentiation, probably by inhibiting FGF-mediated phosphorylation of ERK1/2 .Inhibits TGFB-induced epithelial-to-mesenchymal transition in lens epithelial cells . | [A5D992](https://www.uniprot.org/uniprot/A5D992) |
| 505423 | **LGR4** | Leucine-rich repeat-containing G-protein coupled receptor 4 | G-protein coupled receptor, Receptor for R-spondins that potentiates the canonical Wnt signaling pathway and is involved in the formation of various organs. Upon binding to R-spondins, triggering the canonical Wnt signaling pathway to increase expression of target genes. | [F1MLX5](https://www.uniprot.org/uniprot/F1MLX5) |
| 327681 | **FST** | Follistatin (FS) (Activin-binding protein) | Activin-binding protein, Binds directly to activin and functions as an activin antagonist. Specific inhibitor of the biosynthesis and secretion of pituitary follicle stimulating hormone (FSH). | [P50291](https://www.uniprot.org/uniprot/P50291) |
| 505025 | **ID2** | DNA-binding protein inhibitor ID-2 (Inhibitor of DNA binding 2) (Inhibitor of differentiation 2) | Transcriptional regulator (lacking a basic DNA binding domain) . Implicated in regulating a variety of cellular processes, including cellular growth, senescence, differentiation, apoptosis, angiogenesis, and neoplastic transformation. | [Q3ZC46](https://www.uniprot.org/uniprot/Q3ZC46) |
| 281103 | **CCN2** | CCN family member 2 (Cellular communication network factor 2) (Connective tissue growth factor) | Connective tissue growth factor, Major connective tissue mitoattractant secreted by vascular endothelial cells. Mediates heparin- and divalent cation-dependent cell adhesion in many cell types including fibroblasts, myofibroblasts, endothelial and epithelial cells. | [O18739](https://www.uniprot.org/uniprot/O18739) |
| 538536 | **FZD5** | Frizzled class receptor 5 | amyloid-beta binding,protein-containing complex binding,protein kinase binding,ubiquitin protein ligase binding,Wnt-activated receptor activity,Wnt-protein binding Source. | [F1MGM1](https://www.uniprot.org/uniprot/F1MGM1) |
| 445417 | **FZD1** | FZD1 protein (Frizzled class receptor 1) | frizzled binding,PDZ domain binding,Wnt-activated receptor activity,Wnt-protein binding Source. | [A7MB15](https://www.uniprot.org/uniprot/A7MB15) |
| 616913 | **FZD8** | Frizzled class receptor 8 | PDZ domain binding,ubiquitin protein ligase binding,Wnt-activated receptor activity,Wnt-protein binding Source. | [A0A3Q1MA92](https://www.uniprot.org/uniprot/A0A3Q1MA92) |
| 508008 | **WNT5B** | Protein Wnt | Ligand for members of the frizzled family of seven transmembrane receptors. | [A0A3Q1LVG1](https://www.uniprot.org/uniprot/A0A3Q1LVG1) |
| 326284 | **FGF8** | Fibroblast growth factor (FGF) | fibroblast growth factor receptor binding,growth factor activity Source,type 1 fibroblast growth factor receptor binding,type 2 fibroblast growth factor receptor binding Source. | [F1N6G8](https://www.uniprot.org/uniprot/F1N6G8) |
| 521224 | **LOC521224** | Uncharacterized protein | Uncharacterized protein. GTPase activator activity,metal ion binding,phospholipid binding. | [F1N4Y6](https://www.uniprot.org/uniprot/F1N4Y6) |
| 616742 | **EFNA5** | Ephrin-A5 | chemorepellent activity,ephrin receptor binding,neurotrophin TRKB receptor binding. | [Q17R05](https://www.uniprot.org/uniprot/Q17R05) |
| 281240 | **IGF2** | Insulin-like growth factor II (IGF-II) (Erythrotropin) [Cleaved into: Insulin-like growth factor II; Preptin] | The insulin-like growth factors possess growth-promoting activity,and involved in tissue differentiation. In adults, involved in glucose metabolism in adipose tissue, skeletal muscle and liver. Acts as a ligand for integrin which is required for IGF2 signaling. | [P07456](https://www.uniprot.org/uniprot/P07456) |
| 618481 | **FGF3** | Fibroblast growth factor (FGF) | fibroblast growth factor receptor binding,growth factor activity. | [E1BGY3](https://www.uniprot.org/uniprot/E1BGY3) |

**Table S3 The main function of the proteins encoded by the concerned genes.** The green font indicates the genes with relative decreased level of expression in MT-F7 (MC-F7 as control). The annotations from uniprot (<https://www.uniprot.org/>) exhibited the main functions of proteins in Bos taurus species.

| **Gene ID** | **Gene names** | Protein names (from Uniprot : https://www.uniprot.org/) | Main function( from Uniprot : https://www.uniprot.org/) | **Entry** |
| --- | --- | --- | --- | --- |
| 281181 | **GAPDH** | Glyceraldehyde-3-phosphate dehydrogenase (GAPDH) (EC 1.2.1.12) (Peptidyl-cysteine S-nitrosylase GAPDH) (EC 2.6.99.) | Has both glyceraldehyde-3-phosphate dehydrogenase and nitrosylase activities, thereby playing a role in glycolysis and nuclear functions, respectively. | [P10096](https://www.uniprot.org/uniprot/P10096) |
| 281141 | **ENO1** | Alpha-enolase (EC 4.2.1.11) (2-phospho-D-glycerate hydro-lyase) (Enolase 1) (HAP47) (Non-neural enolase) (NNE) (Phosphopyruvate hydratase) | Glycolytic enzyme the catalyzes the conversion of 2-phosphoglycerate to phosphoenolpyruvate .In addition to glycolysis, involved in various processes such as growth control, hypoxia tolerance and allergic responses. | [Q9XSJ4](https://www.uniprot.org/uniprot/Q9XSJ4) |
| 404148 | **PGAM1** | Phosphoglycerate mutase 1 (EC 5.4.2.11) (EC 5.4.2.4) (BPG-dependent PGAM 1) (Phosphoglycerate mutase isozyme B) (PGAM-B) | Interconversion of 3- and 2-phosphoglycerate with 2,3-bisphosphoglycerate as the primer of the reaction. | [Q3SZ62](https://www.uniprot.org/uniprot/Q3SZ62) |
| 508683 | **PFKL** | ATP-dependent 6-phosphofructokinase, liver type (ATP-PFK) (PFK-L) (EC 2.7.1.11) (6-phosphofructokinase type B) (Phosphofructo-1-kinase isozyme B) (PFK-B) (Phosphohexokinase) | Catalyzes the phosphorylation of D-fructose 6-phosphate to fructose 1,6-bisphosphate by ATP, the first committing step of glycolysis .Negatively regulates the phagocyte oxidative burst in response to bacterial infection by controlling cellular NADPH biosynthesis and NADPH oxidase-derived reactive oxygen species. | [A1A4J1](https://www.uniprot.org/uniprot/A1A4J1) |
| 507476 | **PGK1** | Phosphoglycerate kinase 1 (EC 2.7.2.3) | Catalyzes one of the two ATP producing reactions in the glycolytic pathway via the reversible conversion of 1,3-diphosphoglycerate to 3-phosphoglycerate. | [Q3T0P6](https://www.uniprot.org/uniprot/Q3T0P6) |
| 280808 | **GPI** | Glucose-6-phosphate isomerase (GPI) (EC 5.3.1.9) (Autocrine motility factor) (AMF) (Neuroleukin) (NLK) (Phosphoglucose isomerase) (PGI) (Phosphohexose isomerase) (PHI) | In the cytoplasm, catalyzes the conversion of glucose-6-phosphate to fructose-6-phosphate, the second step in glycolysis, and the reverse reaction during gluconeogenesis .Besides it's role as a glycolytic enzyme, also acts as a secreted cytokine. | [Q3ZBD7](https://www.uniprot.org/uniprot/Q3ZBD7) |
| 282261 | **IGFBP3** | Insulin-like growth factor-binding protein 3 (IBP-3) (IGF-binding protein 3) (IGFBP-3) | IGF-binding proteins prolong the half-life of the IGFs and have been shown to either inhibit or stimulate the growth promoting effects of the IGFs on cell culture. | [P20959](https://www.uniprot.org/uniprot/P20959) |
| 281274 | **LDHA** | L-lactate dehydrogenase A chain (LDH-A) (EC 1.1.1.27) (LDH muscle subunit) (LDH-M) | involved in step 1 of the subpathway that synthesizes (S)-lactate from pyruvate. This subpathway is part of the pathway pyruvate fermentation to lactate, which is itself part of Fermentation. | [P19858](https://www.uniprot.org/uniprot/P19858) |
| 618607 | **AKR1A1** | Aldo-keto reductase family 1 member A1 (EC 1.1.1.2) (EC 1.1.1.33) (EC 1.1.1.372) (EC 1.1.1.54) (Alcohol dehydrogenase [NADP(+)]) (Aldehyde reductase) (Glucuronate reductase) (EC 1.1.1.19) (Glucuronolactone reductase) (EC 1.1.1.20) | Aldo-keto reductase, Catalyzes the NADPH-dependent reduction of a wide variety of carbonyl-containing compounds to their corresponding alcohols. | [Q3ZCJ2](https://www.uniprot.org/uniprot/Q3ZCJ2) |
| 513586 | **MPI** | Mannose-6-phosphate isomerase (EC 5.3.1.8) (Phosphohexomutase) (Phosphomannose isomerase) (PMI) | Involved in the synthesis of the GDP-mannose and dolichol-phosphate-mannose required for a number of critical mannosyl transfer reactions. | [Q3SZI0](https://www.uniprot.org/uniprot/Q3SZI0) |
| 282637 | **CDH1** | Cadherin-1 (Epithelial cadherin) (E-cadherin) (CD antigen CD324) [Cleaved into: E-Cad/CTF1; E-Cad/CTF2; E-Cad/CTF3] | Cadherins are calcium-dependent cell adhesion proteins,and involved in mechanisms regulating cell-cell adhesions, mobility and proliferation of epithelial cells. | [Q6R8F2](https://www.uniprot.org/uniprot/Q6R8F2) |
| 535043 | **ITGA6** | Integrin subunit alpha 6 | insulin-like growth factor I binding,neuregulin binding | [P53712](https://www.uniprot.org/uniprot/P53712) |
| 512975 | **CLDN7** | Claudin-7 | Multi-pass membrane protein, Plays a major role in tight junction-specific obliteration of the intercellular space. | [Q3B7N4](https://www.uniprot.org/uniprot/Q3B7N4) |
| 404153 | **CLDN3** | Claudin-3 | Multi-pass membrane protein, Plays a major role in tight junction-specific obliteration of the intercellular space, through calcium-independent cell-adhesion activity. | [Q765N9](https://www.uniprot.org/uniprot/Q765N9) |
| 281258 | **F11R** | Junctional adhesion molecule 1 (Junctional adhesion molecule A) | integrin binding,PDZ domain binding,protein homodimerization | [A0A3Q1MSB5](https://www.uniprot.org/uniprot/A0A3Q1MSB5) |
| 509566 | **ALDOA** | Fructose-bisphosphate aldolase (EC 4.1.2.13) | Involved in step 4 of the subpathway that synthesizes D-glyceraldehyde 3-phosphate and glycerone phosphate from D-glucose. | [A0A3Q1LMG1](https://www.uniprot.org/uniprot/A0A3Q1LMG1) |
| 504584 | **ALDOC** | Fructose-bisphosphate aldolase (EC 4.1.2.13) | Involved in step 4 of the subpathway that synthesizes D-glyceraldehyde 3-phosphate and glycerone phosphate from D-glucose. | [A0A3S5ZPB0](https://www.uniprot.org/uniprot/A0A3S5ZPB0) |
| 507119 | **PFKP** | ATP-dependent 6-phosphofructokinase (ATP-PFK) (Phosphofructokinase) (EC 2.7.1.11) (Phosphohexokinase) | Catalyzes the phosphorylation of D-fructose 6-phosphate to fructose 1,6-bisphosphate by ATP, the first committing step of glycolysis. | [E1BCW3](https://www.uniprot.org/uniprot/E1BCW3) |
| 281181 | **GAPDH** | Glyceraldehyde-3-phosphate dehydrogenase (GAPDH) (EC 1.2.1.12) (Peptidyl-cysteine S-nitrosylase GAPDH) (EC 2.6.99.) | Has both glyceraldehyde-3-phosphate dehydrogenase and nitrosylase activities, thereby playing a role in glycolysis and nuclear functions, respectively. | [P10096](https://www.uniprot.org/uniprot/P10096) |

**Table S4 Genes play different roles in respective biological process or signaling pathways.** Such as the simultaneous appearance of PFKL, ENO1, PGK1 and GAPDH in Carbohydrate metabolic process, Carbohydrate catabolic process, and HIF 1 signaling pathway.

| **GO/KEGG pathways-DOWN** | **count** | **genes** |
| --- | --- | --- |
| Carbohydrate catabolic process**,**  Carbohydrate metabolic process**,**  HIF-1 signaling pathway | 4 | PFKL, ENO1, PGK1, GAPDH |
| Carbohydrate metabolic process**,**  HIF-1 signaling pathway | 1 | LDHA |
| Carbohydrate catabolic process, Carbohydrate metabolic process | 7 | GPI, PGAM1, ACTN3, FBP1, PPP1R3B, MIOX, AKR1A1 |
| Cell adhesion molecules | 20 | LOC100300510,CDH1,CLDN3,CLDN7,CD58,CLDN4,BOLA-DMB, BOLA-DRA, BOLADOA, NRXN1, CLDN19, ITGA6, IGSF11, SIGLEC1, ICOSLG, CD8B, LOC100848815,F11R,BLA-DQB,ITGA9 |
| HIF-1 signaling pathway | 12 | HK2,PIK3CD,PDK1,ALDOA,EGLN1,PRKCB,PFKP,ALDOC,HMOX1,NOS2,CAMK2B,FLT1 |
| Carbohydrate metabolic process | 9 | PPP1R3C,PHLDA2,IGFBP3,TFF3,MOGAT1,GULO,NPL,MPI,POMC |
| **GO/KEGG pathways-UP** | **count** | **genes** |
| Cell surface receptor signaling pathway**,**  Hippo signaling pathway | 1 | TGFB2 |
| Cell surface receptor signaling pathway**,**  Ras signaling pathway | 3 | PIK3R1,TEK,FGF5 |
| Cell surface receptor signaling pathway | 37 | NDP,RSPO3,TCIM,CDH6,FOS,CTNNBIP1,PTN,LGR5,EDN1,FRZB,SOCS2,ARL2BP,FAIM2,GFRA2,APOA1,ADGRD1,ADRB2,ELAPOR2,SPRY1,PLPP3,GLRB,APOD,ADGRL3,P2RY2,TRADD,PBLD,FST,EID2,CALCRL,MSX2,ISG15,IL18,EYA2,LGR4,ADORA1,MMP9,TMEM17 |
| Hippo signaling pathway | 20 | BMP5,TGFB3,FZD1,SERPINE1,CRB1,LEF1,CTNNA2,CCN2,WNT5B,FZD8,WNT5A,BMP7,ID2,CRB2,WWTR1,FRMD6,APC2,AMOT,BMPR1B,FZD5 |
| Ras signaling pathway | 23 | PLCE1,GAB2,ZAP70,GNG12,IGF2,KDR,LOC521224,RASGRP1,PDGFC,NGFR,PAK3,EFNA5,HTR7,FGF3,GNG11,RASAL2,VEGFC,GNB3,FGF10,FGF8,RGL1,PIK3R3,MAPK10 |
